# Medico-Chirurgical Transactions

**Published:** 1830-01-01

**Authors:** 


### Medico-Chirurgical Review,

### No. XXIII.

OCTOBER 1, 1829, TO JANUARY 1, 1830.

### I.

### A Pathological Inquiry into the Secondary Effects of Inflammation of the Veins. By *James M. Arnott*, Surgeon.

[Medico-chirurgical Transactions, Vol. XV. Part I.]

Many of our readers are probably acquainted with the story of Rosicrusius's sepulchre. That would-be philosopher, and founder of the Rosicrusian sect, having re-invented, or affirmed that he had re-invented, the ever-burning lamps of the ancients, placed one in a tomb, but contrived a piece of clock-work which dashed it into a thousand pieces on the approach of a person who wished to examine it. This desire to monopolize knowledge is not and was not confined to a miserable sect, but cleaves very often to the strongest mind, and has shewn itself in all ages, from those of the Stagyrite to the nineteenth century. Alexander the Great wrote a letter of expostulation to Aristotle on that philosopher's publishing some portion of his writings, and the conqueror of Asia from the Hellespont and Jaxartes to the Indian Ocean and the Ganges was mean enough to complain somewhat peevishly that his instructor had made known to all the world those secrets in learning, which he had before communicated to him in private lectures. Whatever may be the faults of Journalism, to borrow a term from the Polignac vocabulary, it is any thing but Rosicrusian in its spirit or its practice, and if learning was beginning to be deemed a common strumpet in the days of the Spectator, what shall we say to the lady's character now? Thanks to the periodical press, her embraces are enjoyed by the "camp, pioneers and all!" We are not about to enter into general or political considerations respecting the value of periodical literature, for they neither suit our purpose nor our taste, but we may offer a few remarks on our own more peculiar department. We do believe that the medical journals have done and are doing a great deal for the profession, not only in diffusing information but in diffusing the taste for acquiring it; insulated facts are collected, collated, and canvassed, the substance of ponderous and expensive books is laid in a cheap form on the table of every man, the practitioner in the country becomes acquainted with the professional transactions of the town, and in fine the journals become a kind of galvanic medium connecting in some sort the disjecta membra of the body medical. It would also be idle to deny that the periodical press exercises a very material influence on the minds of the profession at large, and that if properly and ably conducted, its powers will be daily augmented. It is well for all concerned when that influence and those powers are directed to their just and constitutional ends—the improvement of their science. When made a vehicle of rancour and personal or party violence, the press becomes a nuisance and a curse to the community which it infests.

Our object has ever been, and so long as we are journalists ever will be to make ourselves as useful to the *state* as our poor abilities will permit, and to do what we deem to be right, no matter how or whom it may displease. Whilst others were engaged in "bloody brawl," we have held on, as far as circumstances permitted, the even tenour of our way, careless of the venomous assault of the gladiatorial desperado, or the seducing blandishments of too aristocratic bodies. The event has proved that we were right, and the sound sense of the members of the profession has shewn them that their real interests are better consulted by cultivating scientific investigations, than by cutting each other's throats, albeit in a literary way.

Having made these few preliminary observations, we shall enter on the more immediate subject of the present article. We are certainly not distinguished by an opposition to the cultivation of pathology, as it is called—quite otherwise; but we have also raised our voices more than once against the pathologic bigots, for they are many, of the present day. The scalpel is an excellent thing in its way, but it is not the Alpha and Omega of medicine, it is not that devilish and charmed elixir, which the alchemists of modern morbid anatomy are vain enough to think it. Before we go farther we shall place on record an opinion of Laennec, as genuine and sound a pathologist as ever graced the annals of our science.

"The study of pathological anatomy," says that great man, "in making us acquainted with the existence of important organic lesions in many cases in which practitioners, too much addicted to the exclusive observation of symptoms, saw only cachexies, or alterations of the fluids, or at least nervous affections, has made us fall gradually into an error of an opposite kind: and among the present race of our pupils, many are as little disposed to acknowledge any nervous diseases besides the organic affections of the nerves, brain, and spinal marrow, as to admit any primary morbid changes in the fluids of the animal body. Nevertheless we are bound to admit that every disease in which we can discover no constant lesion of the solids nor evident alteration in the fluids, must consist in some disorder of the nervous influence."

We may see hereafter whether these remarks apply to any part of the "Memoir on the Secondary Effects of Inflammation of the Veins," which forms the text of this review. No two subjects, perhaps, have attracted more attention within the last few years than inflammation of the veins, and the purulent depôts in internal parts, which follow great operations and injuries:—the hospitals teemed, the societies were bored, and the journals filled with them. On the Continent they attracted nearly as much attention as here, and the several memoirs of Velpeau attest the attention bestowed on these cases in the Perfectionnement and other hospitals of Paris. Those who like every thing by starts and nothing long, grew tired of the investigation and expressed disgust at what they considered and termed the sameness and monotony of the subject, but all who took a genuine interest in scientific questions sympathized with the zeal and perseverance evinced on the occasion, zeal and perseverance which bade fair to throw some light on the difficult subject they encountered. This reasonable expectation has not been deceived, and we now possess a mass of information on what used to be considered by all, and is still considered by many, a most obscure class of affections, which does honor to the labours of the investigators and is highly important to their brethren in practice. No one has distinguished himself more by patient research, ingenuity, and industry than the author of the memoir before us, Mr. Arnott, to whom we gladly pay a tribute hardly earned and justly due. If we find occasion hereafter to differ from him in opinion we shall do so with candour and respect, sensible that though placed in the critical chair we are not on that account to dogmatize and bully, or promulgate our opinions as infallible. The following are the reasons which induced our author to commence his inquiry into the secondary effects of inflammation of the veins.

"A degree of doubt seems still to prevail as to the cause of the alarming constitutional affection frequently attendant on Inflammation of the Veins, and much obscurity unquestionably exists, with regard to the origin of those abscesses and inflammations in distant parts which sometimes occur after injuries. An attempt to remove the one, and to dispel a portion of the other, may not therefore be considered as altogether unworthy of notice.

"My attention was more particularly called to this subject, by some occurrences which marked the course and termination of three fatal cases of inflammation of the veins after venesection, which I had an opportunity of observing. In one of these, a deposition of pus, without signs of previous inflammation, took place under the skin of the opposite fore-arm; in another destructive inflammation of the knee-joint, with a deposition of pus into the cellular substance of the thigh; and in the third, collections of matter at several points in the substance of the lungs; while in all three, the inflammation of the vein did not extend to the heart. These circumstances suggested inferences as to the cause and nature of the constitutional affection in cases of Phlebitis, and views with regard to the origin of abscesses in remote situations arising from injuries, which led to an examination of the evidence on both these subjects, to be found in the writings and observations of others. The results of this investigation I submit to the Society." 2.

Mr. Arnott divides his paper into two parts, the first of which treats of inflammation of the veins, the second of the purulent depositions in the viscera, joints, and cellular texture after injuries, operations, and parturition. An appendix containing a case of phlebitis with a deposition of pus into the substance of the heart, communicated by Mr. Lawrence, concludes the memoir. The mere classification evinces a desire to connect and group together affections that were formerly deemed to be remote, or at all events had no such propinquity stamped upon them. Phlebitis, the traumatic depôts, and phlegmatia dolens will be found by and bye as we deepen in this memoir to be fraternally related in the author's mind. In the first part which treats exclusively of phlebitis Mr. Arnott commences by an abregé of the opinions of authors on the disease, then details seventeen cases collected from various sources, makes observations on those cases, proceeds to the enumeration of the secondary consequences of phlebitis, and finishes by remarks on those sequelæ. We believe that this is as good an arrangement as any that could be adopted, as the facts are first spread before the reader and the inferences that follow are more justly and more readily appreciated. It is the plan adopted by the best practical writers, for although it affords a narrow field for flights of fancy and freaks of imagination, it is more conducive to sober and sound demonstration.

Mr. Hunter remarks that, "in all cases where inflammation of veins runs high, or extends itself considerably, *it is to be expected that* the whole system will be affected. For the most part, the same kind of affection takes place which arises from other inflammations, with this exception, that when no adhesions of the sides of the vein are formed, or where such adhesions are incomplete, pus passing into the circulation may add to the general disorder and even render it fatal."* In referring to the inflammation of the jugular vein in horses which he had often seen, Mr. Hunter professes himself at a loss to determine whether the extension of the inflammation to the heart or the intermixture of the morbid venous secretion with the blood was the cause of death. Mr. Abernethy, who had not seen a case of suppuration in the vein, transcribed the opinions of Mr. Hunter respecting the dangerous mixture of the pus and blood, and likewise stated that when the phlebitis is extensive much symptomatic fever will *probably* ensue, not merely from the inflammation per se, but also, "*because irritation* will be continued along the membranous lining of the vein to the heart."† Mr. Arnott cites this passage as proving that Mr. Abernethy gave a fillip to the doctrine of his master, and assumed as of *actual* occurrence what the other considered as merely *possible*. We think Mr. Arnott has been hasty in his accusation, for Mr. Abernethy after all only rates the extension of the *irritation* by continuity as a *probable* cause of the fatal event; irritation too is not inflammation, and Mr. A. selects the former expression.

Mr. Hodgson affirms that the inflammation extends "in some instances even to the membrane which lines the cavity of the heart," and that the symptoms bear a striking likeness to those of typhus fever.‡ Mr. H. considers that this typhoid type may arise either from the extent of the inflamed surface, or from the intermixture of the pus and blood. Mr. Travers distinguishes the cases where phlebitis ends in the deposition of lymph, and those in which it causes the formation of pus; the former sometimes extending, it is said, to the heart, terminating in a few days with typhoid symptoms, and never in his opinion recovered from; the latter producing hectic ending in exhaustion, but not so fatal as the former. Mr. T. observes,

"'There have been many conjectures respecting the cause of the fatal termination of these cases, at which I confess I feel surprised; among others, the inflammations by extension of the heart, or the membranes of the brain, and the conveyance of pus into the circulation, have been mentioned. Not to insist on the innocuous quality of pus, it should be observed, that the most rapidly destructive inflammation is that which has the true adhesive progress in which no pus is secreted. But if we consider the importance of the veins in the economy, the extent of surface which the collective areæ of the venous trunks afford, larger, I imagine, than any of the shut sacs of the body, and the diffused and disorganizing character of the inflammation, we can surely be at no loss to account for the disturbance of the system. It is an error to suppose that any quicker sympathy exists between the constitution and the venous, than the ar-

---

* Trans. of a Soc. for the Improvement of Med. and Chir. Knowledge, Vol. I. London, 1793, p. 18.

† Essay on the Occasional Ill-consequences of Venesection. Surgical Works, Vol. II. London, 1823. p. 150.

‡ On Diseases of Arteries and Veins. London, 1815. p. 511, et seq.

terial or absorbent system. I say this, because I have observed something like that superstitious alarm which is excited by events that we do not expect, and cannot explain, has been produced by the fatal catalogue of tied veins, and a comparison of this with the generally successful cases of tied arteries. All the mystery of veins is, as I have attempted to show, that they are indisposed to inflame, but when excited, inflame by continuity, and *therefore* it is that the constitution sympathizes so deeply.' "* 6.

Who can read this passage without seeing how words are erected into things, and the expression of certain facts in other language offered as the obvious *explanation* of those facts? The marrow of the whole is this;—that phlebitis is fatal, the extent of inflamed surface is great, the inflammation severe, and the constitution much affected *on account* of veins inflaming "by continuity." The disclosure leaves us much where we were before.

Mr. Carmichael cleaves to the extension of inflammation "to the cava and perhaps to the heart," and the mingling of the blood and pus.† M. Breschet connects typhus and phlebitis within the cranium as effect and cause, but leaves the *how* to the imagination of his readers.‡ M. Bouillaud holds to the presence of pus in the system, and flanks his views by the experiments of Baglivi, Majendie, and Gaspard on the injection of acrid and putrid matters into the veins of animals.§ M. Ribes throws out the opinion that the veins and venous blood are primarily affected in the fievres adynamiques, but like M. Bouillaud he has left the *quomodo* of the connection between the local and constitutional effects of phlebitis unessayed.||

Mr. Guthrie who has treated of phlebitis after amputation "assumes" that it is adhesive or healthy, and easily cured; erysipelatous or unhealthy, and almost invariably fatal. He states the facts and leaves the explanation to those who can give one.¶ Sir A. Cooper asserts the occasional extension of inflammation to the large veins and heart, and remarks that if pus remains in a vein without pointing, life is destroyed by excessive constitutional irritation.**

The above are the explanations or theories advanced by authors from Mr. Hunter down to Sir Astley Cooper to account for the fatality of phlebitis. Mr. Arnott is not satisfied with all or any of them, and thinks that the doubt which yet prevails respecting the true rationale of the case is rather attributable to the question not having hitherto received the careful attention requisite to solve it. Mr. Arnott proceeds to the detail of cases in order to fulfil the desideratum he deplores, and our readers will presently see with what success.

### Fatal Cases of Inflammation of Veins.

The first three cases were witnessed and are detailed by Mr. Arnott him-

---

* Cooper and Travers' Surgical Essays, vol. I, 3d edit. London, 1818, p. 286.

† Trans of King's and Queen's Coll. of Physicians in Ireland. Vol. II. Dub. 1818, p. 355, et seq.

‡ Journ. Complement. Tome II, p. 325, et tome III, p. 317. Paris, 1819.

§ Révue Medicale, Juin, 1825. p. 424,5.

|| Mémoires de la Société Médicale d'Emulation. Tome 8, Paris, 1817, p. 624.

¶ On Gun-shot Wounds, &c. 3d. edit. 1827, p. 299.

** Lectures on Surgery, by Tyrrell. Vol. III. London, 1827, p. 205 et seq.

self, the remainder have been collected with much industry from various sources. Ten of the following instances of phlebitis are from venesection, two from the operation for aneurism, three from amputation, and two from excision and division of the saphena.

*Case* 1. *After venesection.* Sophia Brancher, æt. 25, was bled on the 27th Nov. 1826, from the left median basilic vein for an unimportant accident received on the preceding day. On the 28th she resumed her occupation of weaving, and towards evening the arm felt stiff and painful. On the 29th, she continued at her work, and all the symptoms became aggravated; on the two following days she was unable to use the arm which she poulticed, and on the 2nd of December she entered the hospital. At this time a small crust covered the wound made in venesection, and the inside of the arm above and below the elbow joint was swollen, red, and very painful upon pressure. The face was pale and anxious, the skin hot and dry, the pulse full and 120, the tongue white and moist, great thirst, no appetite, bowels open from medicine. Sixteen ozs. of blood abstracted from the opposite arm induced fainting; it was highly cupped and buffed. She passed a bad night and on the 3d complained of pain in the abdomen, increased by pressure or deep inspiration. This pain was removed by leeches, and on the 5th the arm was easier, but the inflammation was extending towards the axilla. 7th. Arm less inflamed and wound in the vein discharging freely pus sometimes mixed with blood;—face very pale and anxious, pulse small and 104, tongue furred and dry in the middle, pains throughout yesterday and to-day all over the body, especially in the extremities. On the 8th, the respiration was hurried, and the pains very severe in the calves of the legs; on the 9th she was better, but the countenance was more anxious and sallow; on the 10th the improvement was still more marked; on the 11th the left arm had nearly regained its natural size, but she complained much of the pains in her limbs, and towards evening the respiration grew laborious, the skin cold, the bowels were much purged and the abdomen painful upon pressure. 12th. Face pale and altered, matter has formed under the skin of the *right* fore-arm without cutaneous redness and five ounces have been evacuated by puncture, the left knee is swollen and painful from effusion into the joint. She now sank rapidly and died early on the morning of the 14th.

"The husband chose to be present at the examination after death, to restrict it in point of time and extent: it was therefore hurried and imperfect. The following points were, however, ascertained. Inflammatory condensation of the cellular tissue of the fore-arm, and arm in the inflamed part. A chain of small suppurations *in the course of the blood-vessels*, with white healthy pus from the elbow to the axilla. The axillary and subclavian veins, the superior cava, and the lining of the heart were quite natural. No diseased appearances in the chest; the liver light coloured, and beginning to assume the yellow appearance produced by indulgence in spirits. The other abdominal viscera were sound.

"The knee was not allowed to be examined." 13.

The account of the dissection is indeed imperfect for no mention is made of the state of the vein which had been inflamed in the first instance. It is worth remarking that although the respiration was laborious and the abdomen painful upon pressure no diseased appearances were found in either cavity.

*Case 2, after Venesection.* John Carr, æt. 47, was bled Jan. 1st, 1827, for a strain in his back, and returned to the hospital next day with pain in the wound and inflammation of the cellular tissue. The face was pale and anxious, the pulse frequent full and hard, and he had pain in the chest on taking a full inspiration. On the 3d, the pulse being full, hard, and 116, he was bled to 20 ounces, when he fainted; the blood was much buffed and the crassamentum very tough. On the 4th the pulse was full, hard, and upwards of 100, in consequence of which he was bled again to 20 ounces with deliquium, and the blood was again highly cupped and buffed whilst the serum had a milky appearance. He breathed easier in the evening, and next day the thoracic symptoms were nearly gone. 6th. Arm more swollen, pus discharged on pressure from the orifice of the vein, purging, shivering fits in the evening. 7th. Countenance pale and anxious, great irritability, pulse small and 140, tongue slightly brown and dry. On the 10th, he was in much the same state, save that the respiration was laborious and the countenance sallow; the inflammation in the course of the vein was diminished, but the discharge from the orifice was greater and mixed with bubbles of air. On the 13th the inflammation was increasing towards the axilla, and on the 18th fluctuation being felt near the insertion of the deltoid, a puncture was made and two ounces of thick, yellow, putrid purulent matter let out. On the 20th he complained of pain in the chest, pulse small and 104, wound in the vein discharging no pus, abscess in the axilla healing up. 25th. After taking cold drink last night was attacked by a severe rigor, and during the night and this morning he has had several more; he is very low, and the cough is accompanied with copious purulent expectoration. Delirium supervened, the irritability was great, the breathing so difficult at times as to threaten suffocation and then accompanied with a peculiar cry. The exhaustion made progress, the suffocative paroxysms became more frequent, and he died at 6, a. m. of the 30th.

*Sectio Cadaveris, Six Hours after Death.* " An incision was made from the clavicle to the middle of the fore-arm, so as to divide the skin, which was dissected back. A small abscess, with a smooth secreting surface, was found between the basilick and median basilick veins, and opposite the external wound. Both these veins were impervious to blood, and were reduced to a cord-like substance. On tracing the basilick-vein upwards, it was found to terminate in an oblong shaped abscess about two inches above the original wound in the vein: it had a thick and irregular smooth surface, and the surrounding cellular tissue was very dense; the basilick proceeding from the abscess was lined with an irregular false membrane for about two inches, when it terminated in a quantity of fibrin, which filled up the cavity of the vein. Immediately above this, the cephalick and several smaller veins terminated. The internal surface of the heart and of the large veins appeared healthy, and were filled with recently coagulated blood.

" The pericardium contained a few ounces of a yellowish serum mixed with portions of lymph. On the surface of the heart several large white spots appeared. There were extensive adhesions between the pleura costalis and pulmonalis. The lungs were healthy, excepting several points where *vomicæ* had formed; a very large one was situated in the posterior part of the right lobe.—The bronchiæ were filled with mucus mixed with air.

" The abdominal viscera were healthy.

" The arachnoid membrane, covering the hemispheres of the brain, was thickened, opaque, and of a milky appearance Serous fluid was effused into the texture of the pia mater. Several ounces of yellowish serum were found in the lateral ventricles. The substance of the brain was healthy." 18.

We beg the reader to observe, that the abscesses in the lungs are described as *vomicæ*, a very different kind of thing from the genuine traumatic depôt. And yet Mr. Arnott farther on cites this case of Carr amongst the instances of "hepatization of the lungs, the infiltration of pus into their tissue, or small collections like a mixture of pus and lymph." The appearances too of the arachnoid and pia mater, which will by-and-bye be found ranked in the sequelæ of phlebitis by Mr. Arnott, appear to us to be extremely common, and by no means to deserve the importance into which they are elevated by our author. We shall return to this point hereafter.

*Case 3, after Venesection.* Henry Arnold, æt. 51, admitted Jan. 19th, 1827, with old ulcer of the leg, was twice bled, and three days after the second bleeding the wound of the vein became painful. On the 29th, (sixth day) he had rigors followed by heat and thirst, and on the 31st the inflammation extended from the elbow to the axilla, and he had another rigor, which continued to recur at intervals, until the 4th of February, when he complained of severe pain and some swelling of the left knee-joint. Arm easy, and a little thin pus flowed from the wound in the vein on pressure, countenance yellowish, pulse hard, full, 100. On the 5th, the knee was distended to the utmost with fluid, the thigh swollen, painful, slightly red and very hot, the superficial veins of the knee and thigh excessively distended, the pulse soft and 140. On the 6th, he complained of pain in the right shoulder without swelling or redness, and on the 8th he died.

*Sectio Cadaveris ten hours after death.* "The cephalick-vein, which had been punctured, was thickened, and contained pus for about two inches below, and four inches above the wound, where a coagulum of blood was found filling the cavity; above and below these points the vessel was healthy.

"The arachnoid membrane was thickened, opaque, and whitish. The cellular texture of the pia mater was loaded with serum, and an increased quantity of fluid was found in the ventricles.

"The cavity of the knee-joint was filled with tolerably thick pus of an uniformly reddish colour, as if from an intimate admixture of blood. The synovial membrane was thickened, with an irregular and almost villous surface; it was extremely vascular in its whole extent. The cartilaginous coverings of the femur and tibia had undergone considerable absorption, so that the convexities of the femoral condyles, and the corresponding excavations of the tibia, were completely bare. The cellular substance covering the capsule of the knee, under the extensor muscles, was inflamed, thickened, and loaded with pus. This texture was in the same state on the surface, and throughout the whole substance of the vasti and cruralis muscles. Sections of these muscles presented a most singular appearance, their large fasciculi being separated, apparently, by layers of thick yellow pus. The matter, although precisely similar in colour and consistence to that produced by phlegmonous inflammation, was nowhere collected into an abscess; but was diffused through the cellular structure, as serum is in the case of anasarca. In the rest of the limb there was an effusion of a bright light yellow serum.

"The cellular structure, exterior to the orbicular ligament of the right shoulder, was filled with thick yellow pus; but the cavity of the joint and the deltoid muscle were natural." 20.

This completes the series of three cases observed by Mr. Arnott himself at St. Bartholomew's Hospital. The remainder are from various sources, and we shall endeavour to deal with them as briefly and succinctly as the occasion will permit, for the constant reference of the author to particular cases in his general remarks will prevent us from *skipping* any one.

*Case* 4, *after Venesection.** Gaspar Goldinger was bled in the right arm on the 16th November for epilepsy, and on the following day the puncture was inflamed. 18th. Arm very painful and swollen from below the elbow to the shoulder, face and body yellowish, pulse quick and feeble. The fever and prostration increased, and on the 22d the respiration was short and accompanied with pain in the right side of the chest. He died in the night of the 23d, seven days after the bleeding.

*Sectio Cadaveris.* Cephalic filled with pus from its termination in the axillary to its origin at the bend of the elbow from the median cephalic and superficial radial, the latter of which also contained pus for two inches. Coats of the vein thickened, indurated, and red. Cellular tissue of right pectoral muscle infiltrated with thick greenish pus. Eight or ten ounces of yellowish opaque serum in the right sac of the pleura, adhesions of the pleuræ on the left by a delicate false membrane, small hepatized portions gorged with fluid, puriform in some, in both lungs. Arachnoid membrane opaque and indurated, with fluid over and in the pia mater; some yellowish serum in the ventricles.

*Case* 5, *after Venesection.*† A robust soldier was bled for ophthalmia and next day the arm inflamed. Fever followed, and although the wound in the vein had healed his pulse on the 17th day was 120 and feeble, skin hot, tongue covered with brown fur, respiration difficult, prostration great. On the 23d day a painful swelling was observed above the clavicle, and in a few days a soft diffused swelling underneath the angle of the lower jaw. In the course of the 7th week he died.

*Sectio Cadaveris.* Coats of the cephalic vein thickened and its cavity obliterated from an inch above the puncture to the shoulder—*brachial, axillary, subclavian and internal jugular veins enlarged, thickened, and indurated* —external jugular and subclavian filled with pus, thickened, and lined with lymph—diseased appearances terminating abruptly. A serous fluid with flakes of lymph was contained in the thorax, and the lungs presented some small abscesses. Veins of the pia mater turgid—more serum than usual in the ventricles.

*Case* 6, *after Venesection.*‡ Hugh Johnson, æt. 33, bled on the 15th May, 1810, for ophthalmia. On the 16th the wound inflamed, afterwards discharged pus, and was followed by fever. The wound healed entirely by the 28th, but the fever assumed the decided typhoid type, and the patient died on the 17th day after the operation.

*Sectio Cadaveris.* Wound in the median basilic closed—pus occupying the mediana longa for about two inches below the origin of the median basilic and cephalic—pus along the whole of the humeral vein to the axilla,

* M. Le Heriné, Journ. de Medecine, T. 12, p. 417. Paris, 1806.

† Hodgson, p. 512. ‡ Cooper and Travers' Essays, Vol. I. p. 229.

with an irregular deposit of lymph adhering to its lining membrane—vein abruptly assumed a healthy appearance about an inch before passing under the clavicle—heart healthy, save a small patch of lymph on its anterior surface, and another on the opposite surface of the pericardium—a little fluid in the pericardium and in both sides of the chest.

*Case* 7, *after Venesection.** Michael Dogherty, æt. 31, bled in the right median cephalic on the 15th April, 1821, for continued fever. Inflammation of the vein took place, accompanied with low fever, and on the 5th day pus mixed with blood could be pressed from the wound. On the 10th day had a severe rigor followed by delirium, and pain across the chest. On the 27th day the wrist of the opposite arm was much swollen and presented a distinct fluctuation, whilst exquisite pain was felt in the left knee. On the 30th day he died.

*Sectio Cadaveris.* Coats of the mediana longa greatly thickened, and its inner surface lined with lymph, which cabin'd in a small abscess that had formed about the middle of the fore-arm.

"Two inches above the bend of the arm, the cephalick and basilick veins were filled with pus, and their coats were uncommonly thin, and easily ruptured. This appearance extended to the axillary vein, and terminated abruptly before the vein crossed the first rib. The vein remained quite pervious, though its cavity was much diminished; neither did it contain a particle of blood, nor could its valves be observed.

"Between the first and second ribs, near to their sternal extremities, an opening was discovered leading to a sack, formed by an adventitious membrane of coagulable lymph, filled with purulent matter, and pushing towards the pleura costalis. The sack contained four or five ounces of pure pus. At the left wrist there was a similar collection of matter, containing about six ounces of pus. The lungs were perfectly healthy in their structure, but several old adhesions existed between the pleuras in both sides. The heart was natural and healthy, but on the left side there was a deposition of coagulable lymph on its external surface, and the inner coat of the aorta to its arch had a deep red appearance. The other cavities were examined, but nothing of any importance was found." 26.

*Case* 8, *after Venesection.*† Clementine, æt. 20, 6 months gone with child, bled on the 12th January, 1829. Inflammation of the veins took place, succeeded by fever, and on the 18th a small quantity of pus was evacuated by enlarging the wound. The local symptoms abated but the prostration, &c. increased, and in this state labour came on, when she died two hours after delivery, on the 14th day from the venesection.

*Sectio Cadaveris.* Coats of median basilic thickened, and cavity almost obliterated—veins opening into basilic trunk filled with white healthy-looking pus—coats of brachial vein greatly thickened and lined with concrete membrane, cavity also filled with thin reddish pus, till near the clavicle —inflammatory changes rapidly diminishing from axillary vein onwards, *so that opposite the scaleni the subclavian was not affected*—superior cava and heart healthy—marks of recent pleurisy and pulmonary *engouement*, but not pneumonia.

---

* Trans. of Med. and Chir. Soc. of Edinb. Vol. I. p. 485-6.

† Archives Générales, Août, 1827, p. 503.

*Case* 9, *after Venesection.** Capt. L. æt. 34, bled from the arm on the 3d day after lithotomy. Inflammation attacked the vein, and he died at the end of the third week, having suffered for the last 48 hours from symptoms of pleurisy on the same side as that upon which he had been bled.

*Sectio Cadaveris.* Basilic vein up to its termination and veins corresponding to it down to the back of the hand thickened by inflammation—coats of vessels red, roughened by lymph on their interior, and containing pus—axillary vein and its continuation onward healthy—whole subcutaneous tissue of the arm inflamed, and partially infiltrated with serum. Pleura much inflamed, and about a pint of whey-like fluid mixed with pus and flakes of lymph in its cavity.

*Case* 10, *after Venesection.*† Thomas Fuller, æt. 21, a prize-fighter, bled twice from same aperture in the right median cephalic for anasarca and dry cough, on the 18th June, 1827. On the 21st, the wound inflamed ; on the 22d there were rigors and bilious vomiting ; on the 23d a sero-purulent discharge continually oozed from the wound. The wound was enlarged, and serum, pus, and blood discharged ; hæmorrhage took place in the evening, and another in the night, which was stopped by pressure. The body became yellow, he had no dyspnœa nor cough, and he died in the evening of the 6th day from the venesection.

*Sectio Cadaveris.* A pint of discoloured serum in the right side of chest and old pleural adhesions—same appearances, but slighter in the left—lower lobes of both lungs, particularly the right, fleshy and consolidated—bronchial mucous membrane injected—more water than usual in the pericardium—general disposition to fluidity of the blood and *staining* of the heart and aorta—liver and kidneys enlarged. Median cephalic greatly thickened, as was the cephalic up to the insertion of the deltoid—interior of vein inflamed *up to within two inches and a half of its junction with the axillary*, beyond which it was sound—no pus in the vein—cephalic trunk inflamed on its inner surface below its junction with the median cephalic, and its cavity in one part plugged up with lymph—no obstruction above the puncture—median basilic thickened and inflamed—basilic also slightly affected.

This concludes the cases of phlebitis from venesection, and we now pass on to those which followed the operation for aneurism.

*Case* 11, *after the Operation for Aneurism.*‡ John White, æt. 28, operated on for popliteal aneurism Nov. 29th, 1826, when the femoral vein was slightly wounded and tied by nipping up its coats. On the 9th December, the ligature came away from the vein and a little hæmorrhage succeeded on the 10th, with tenderness in the course of the vessels to the groin. On the 14th there was head-ache ; on the 20th a severe rigor ; on the 21st a slight one, and on the 25th and 26th there was hæmorrhage from the wound. From the 27th the rigors were frequent and severe, and on the 31st he died, thirty-two days after the operation.

---

* Med. and Chir. Trans. Vol. XIV. p. 284.

† Lond. Med. Gaz. Vol. II. p. 155.

‡ Cooper and Travers' Essays, Vol. I. p. 243.

*Sectio Cadaveris.* Communicating with the external wound was an opening in the vein, the coats were thickened and lined internally with adhesive matter. The adhesive inflammation *extended as high as the bifurcation of the cava*, which was also inflamed but contained neither lymph or pus. Considerable serous effusion into the chest—one lobe of left lung covered with recent lymph, and parenchyma of the lungs apparently inflamed—slight inflammation on surface of intestines.

*Case* 12, *after Operation for Aneurism.** James Boyle, æt. 40, operated on for popliteal aneurism, May 20th, 1818, when a gush of venous blood followed the passage of the aneurism needle, but the hæmorrhage ceased spontaneously. On the 5th day a little pus flowed from the wound and there was general *malaise*, increased on the 6th. On the 13th day several distinct rigors, with much depression; another rigor on the 14th; three on the 15th with delirium and prostration. Blood was twice abstracted and found to be buffed, but the patient got worse, had another rigor on the 17th day and died on the 19th day from the commencement of the phlebitis and 24th from the operation.

*Sectio Cadaveris.* Crural vein had been wounded but not tied—its inner surface lined with pus and organized lymph as low as the ham, where the vein became suddenly impervious from deposited lymph—disease extended a considerable way down the saphena, and *upwards as far as the common iliac*, beyond which the examination was not allowed to be prosecuted.

The next three cases exemplify phlebitis after amputation.

*Case* 13, *after Amputation.*† John Crute, æt. 30, had the thigh amputated for scrofulous disease of the knee-joint—was very low for the next two days—suffered on the third‡ from unusual constitutional irritation, and was attacked that night by bilious vomiting—had slight rigors in the evening of the fourth day—prostration and delirium succeeded—and the patient died on the sixth day from the amputation.

*Sectio Cadaveris.* Ligature on the mouth of the femoral vein, *from which along the iliacs and cava to the entrance of the emulgents* the interior was coated by large flakes of lymph—marks of diffused inflammation extending to right auricle.

*Case* 14, *after Amputation.*§ Eliz. Mitchell had the thigh amputated on the 13th of Feb. 1818, for compound dislocation of the ankle-joint which had happened on the 27th of January. In the night of the 17th she vomited, complained of much depression, and next day a previous yellow suffusion was much increased. On the 20th she had a long and violent rigor, many of which continued to recur and she died on the 14th day after the operation.

*Sectio Cadaveris.* Surface of stump gangrenous—femoral vein full of pus as high as Poupart's ligament, coated with soft lymph above this to the cava which was not inflamed—liver sound—examination carried no farther.

---

* Trans. of Coll. of Phys. in Ireland, vol. II. p. 350.

† Cooper and Travers' Essays, vol. I. p. 247.

‡ Mr. Arnott says "eleventh"—an evident mistake.

§ Trans. of Coll. of Phys. Ireland, vol. II. p. 365.

*Case* 15, *after Amputation.** Jane Strangemore had the thigh amputated Sept. 27th, 1823, for disease of the knee-joint—vomited bilious matter next evening—wound had nearly healed on the 8th of Oct.—complained of pain in the other calf and heel on the 9th, which parts swelled and were tender on the 10th, when she again vomited bile, and continued to do so at intervals—was slightly jaundiced on the 25th—died on the 27th, thirty days after the amputation.

*Sectio Cadaveris.* Stump sloughy as high as Poupart's ligament—adhesive inflammation within the vein to this point, above which it contained pus, lymph, and blood along the cava to beyond the diaphragm—traces of inflammation distinctly observed almost into the auricle—inflammation along the left iliac vein into the pelvis and along the femoral as far as the foot—the vein at the left groin distended with pus—viscera healthy.

The two succeeding and concluding cases of venous inflammation were after operations on the saphena.

*Case* 16, *after Excision of Part of a Varicose Vein.*† John Dodging, æt. 35, had a portion of a varicose branch of the posterior saphena excised from the right leg on the 25th June, 1828. Next day inflammation appeared along the thigh, and he had vomiting of greenish matter. On the 28th, dyspnœa, great sickness, and pain in epigastrio—on the 30th, phlegmonous erysipelas of the left arm—on the 2d July pain in the head, brown tongue, and depression—on the 3rd, the patient deeply jaundiced, and evidently sinking, cornea of both eyes opaque, and conjunctiva injected—on the 4th he died.

*Sectio Cadaveris.* Posterior saphena contained lymph and pus as high as the ham where the inflammation terminated abruptly—several small muscular branches contained fluid pus. Deep-seated abscesses beneath the fascia and amongst the muscles of the left fore-arm and leg—sero-purulent effusion between the muscles of the right fore-arm. A small abscess, evidently from recent inflammation in the superior lobe of the right lung. Considerable effusion into the cellular tissue of the pia mater, particularly towards the basis cranii—lymph around the trunks of the carotids—nerve of the third pair on the left side flattened and softer than the right—nerve of the fifth pair on the right side similarly changed, but to a greater extent—crystalline humor of the right eye so soft as to yield to the slightest touch—vitreous humor of a reddish-yellow colour, and its membrane traversed by red vessels—retina of deep red colour.

*Case* 17, *after Division of the Saphena.*‡ A middle-aged man had the saphena major divided where it passes over the internal condyle, on account of varix and ulcer. At 5, a.m. of the 2d day had a shivering and was light headed; sickness at noon. On the morning of the 3d day was sick and much depressed; some redness of the thigh but little pain or tenderness—in the evening the redness, &c. had extended upwards in the course of the saphena major—he died at 3, a.m. of the 4th day.

*Sectio Cadaveris.* Small quantity of matter in external wound—extre-

---

* Med. and Phys. Journal, vol. LVI. p. 35.

† Lond. Med. Gaz. vol. II. p. 284.

‡ Hodgson, London, 1815, p. 555.

mities of saphena divided in the operation united by coagulable lymph—saphena from the wound to its junction with the femoral reddened and vascular, but containing neither lymph nor pus—vena cava and abdominal viscera healthy.

In order to render the account of these cases complete and to preserve the *continuity* of the subject, we shall introduce in this place, a case of phlebitis communicated by Mr. Lawrence and placed in the Appendix of our author's paper.

*Case* 18, *after Venesection.* A. B. æt. 34, received in Bethlem Hospital, Nov. 12, 1828, for mental derangement which had existed fourteen days. He had been bled in the median cephalic and basilic veins, and the right arm from the middle of the fore-arm to the axilla was red, swollen, and painful, with induration in the course of the cephalic and basilic. There was low fever which became complicated with dyspnœa, cough, mucous expectoration, and pain in the epigastric region. He died in the night of the 25th, the redness, swelling, &c. of the limb having quite disappeared for some days before death.

*Sectio Cadaveris.* Subcutaneous tissue at the bend of the elbow and neighbouring part of the arm and fore-arm infiltrated with serum and lymph—median cephalic converted into an impervious cord, as was the cephalic half way up the arm *where it suddenly became healthy*—a little pus about the puncture in the integuments—median basilic and other small branches impervious—basilic thickened, indurated, but containing nothing—axillary and subclavian less thickened but their interior furred with brownish lymph, as far as the termination of the latter which was blocked with a soft mass of lymph—cellular tissue round the axillary and subclavian veins indurated and axillary glands enlarged. Six or seven ounces of opaque yellowish-brown fluid in the pericardium, which was injected, inflamed, and presented portions of ecchymosis—bright yellow patch as large as a sixpence on the surface of the left ventricle—muscular substance of this ventricle softened and partially broken down, with yellow pus diffused amongst the broken texture. Small portion of right lung impervious from recent inflammation—two contiguous lobes partially agglutinated by pleural adhesions. Mucous lining of trachea and bronchial tubes inflamed—liver partially tuberculated on its surface—gall-bladder strongly adherent to neighbouring viscera.

Mr. Lawrence seems to think it strange that the left side of the heart was affected rather than the right. Had the *internal* lining membrane been affected there would be some grounds for surprise, as this membrane is continuous on the right side of the heart with the venous tunics. The suppuration, however, being in the *muscle*, and connected with obvious inflammation of the pericardium, we see no more matter for astonishment than a similar deposition in a particular part of any other muscle would create.

*Case* 19, *after Venesection in the Horse.* A horse was bled in the jugular vein on the 6th of October by a farrier, suppuration took place in the wound, and on the 19th day from the operation the animal died. The vein contained pus above the puncture, and was thickened; it was obliterated for three inches below. Heart natural—depositions in the lungs of a substance like dirty tallow or adipocire—some pleurisy—blood in the stomach.

"From these seventeen cases of fatal Phlebitis, the first conclusion deducible is, the total disproval of the assertion, that death results from the extension of the inflammation of the vein to the heart. In none of the ten instances following venesection was the superior cava affected, much less the heart; and in half this number, inflammation had not reached to the subclavian, or even to the axillary vein. In the cases where the inferior cava had become inflamed, viz. those of Crute, White, and Strangemore, the first is the only one in which the heart is represented to have been actually implicated: and here, the deposition of lymph terminating at the entrance of the emulgent vein, the observation is, that 'there were marks of diffused inflammation extending to the right auricle of the heart, but the signs of adhesive inflammation terminated as described.'

"As a cause of death, then, the extension of the inflammation to the heart may be considered as a mere matter of assumption, the history of the error being simply this: Mr. Hunter's observations not having enabled him to form a decided opinion as to the cause of death, when this affection occurred in the jugular vein of horses, he suggested as a query, whether it might not depend on the inflammation extending to the heart. By succeeding writers this suggestion was adopted, without examination, and without any evidence being offered in its support. Indeed the circumstance itself is of very unusual occurrence; for, with the exception of the instance just alluded to, I have only found two others in which it is alleged, that the inflammation had extended from the vein to the heart, and in these the description is not very precise. Both cases are mentioned by M. Ribes, in the Revue Médicale for July 1825. In one, occurring so far back as the year 1799, where the veins of the arm were inflamed, in connexion with gangrene of the hand from chilblain, 'traces of inflammation' are stated to have been continued into the superior cava, and even to the interior of the right auricle and ventricle; and, in the other instance, where the saphena evinced some signs of inflammation, in a case of mortification of the leg and foot, it is stated in the same vague terms, that 'the right auricle and ventricle of the heart, as well as the inferior cava, at its insertion into this organ, had manifest traces of recent inflammation.'

"It is to be regretted that M. Ribes has not distinctly specified what the 'traces' were, which he considered as indicative of inflammation in the lining membrane of the heart; an omission, which when contrasted with his immediately preceding minute description of the changes produced by inflammation in the coats of the veins, leaves us in considerable doubt as to the comparative value of these traces as proofs of inflammation. Of course, it is not by these remarks meant to be denied, that inflammation may extend to the lining membrane of the heart from the veins; but considering the fact itself as not yet sufficiently established, it is perhaps not requiring too much that the evidence adduced in its support should be of an unequivocal character.

"As the preceding remarks have indicated, there are considerable differences in the extent of vein occupied by inflammation in fatal cases of Phlebitis. Sometimes the disease has spread into several, or most of the veins of a limb from that primarily affected; at others, it has not proceeded beyond the vessel in which it originally appeared. This last circumstance, with that of the fatal consequences sometimes ensuing from inflammation, limited to a few inches only of a vein, (as of about six of the cephalic, in the case of Arnold,) justifies the inference that the dangerous consequences from Phlebitis bear no direct relation to the extent of the vein which is inflamed." 44.

We agree with Mr. Arnott on the great infrequency of the extension of inflammation to the heart, an infrequency which is fully and fairly established by the cases here brought forward. Like Mr. Arnott too, we are inclined to view with scepticism descriptions like those of M. Ribes, where the reader is vaguely told that "traces of inflammation" existed in the vena cava or other great vessels. Nothing is more common in phlebitis than a preternatural fluidity of the blood after death, and a consequent *staining* of the lining membrane of the heart and large vessels, an appearance which might readily deceive those who are not aware of the circumstance. The finest specimen of this condition of the blood-vessels in the Museum of Great Windmill Street was obtained from the body of a man who died of phlebitis in St. George's Hospital. Unless the appearances of inflammation are distinctly specified, no reliance whatever should be placed upon the statement.

"In endeavouring to ascertain the nature of the connexion between the primary and secondary affections in this disease, the question which next suggests itself is, whether the latter depends, as has been alleged, upon the entrance of pus into the circulation. We are thus led to inquire into the contents of the inflamed vessels, and on referring with this view to the cases which have been adduced, it will be found that in a number of them, where an open wound existed in the vein, pus was discharged from it during life. Whilst in fourteen cases out of seventeen, (those of Brancher, Carr, Arnold, Goldinger, that by Mr. Broughton, Johnson, Dogherty, Clementine, Captain L., Fuller, Boyle, Mitchell, Strangemore, and Dodging,) pus, or pus in conjunction with lymph, was present in the vessel after death. In two instances, no mention is made of pus, the contents of the veins being described in the one (White) as 'adhesive matter,' in the other (Crute) where the cava was concerned, as 'flakes of lymph.' In one case only, (Mr. Hodgson's) where the inflammation occurred in a vein previously diseased, or in a vein the branches of which at least were varicose, neither pus nor lymph was found in the vessel.

"It results from this statement, that although pus is present in the veins in the great majority of fatal cases of Phlebitis, and that although it should appear from the character of the general symptoms, and the effects produced upon animals, by the injection of a similar fluid into their vessels, that the passage of pus into the circulation is probably the principal, yet the circumstances do not justify us in regarding it as the sole cause of the secondary affection. In addition to the presumed absence of pus in two instances, and to its declared absence in a third, it may be remarked, that the early appearance of the symptoms in some cases seems scarcely to correspond with the time usually required for the production of pus, as in one which occurred to Mr. Freer, where they came on, suddenly, four hours after ligature of the saphena. If then, the constitutional affection in Phlebitis is to be explained by the introduction of a fluid into the circulation, which contaminates the blood, and operates as a poison, this property must be attributed to inflammatory secretions generally from the vein, although not purulent; and it remains to be seen whether the symptoms of this affection are such as can be accounted for by the passage of pus only into the system." 46.

This question of the contamination of the system in phlebitis by pus or any other morbid secretion from the venous tunic is deeply involved in obscurity and difficulty. The typhoid character of the symptoms, the yellow or sallow skin, the disposition to local inflammations and deposites as well as to preternatural fluidity of the blood after death would appear to point out a contamination of the fluids. But on the other hand, such cases as that of Mr. Freer must stagger the votaries of the *humoral* doctrine. It is certain too that the free passage of pus from the inflamed vein into the general current of the circulation is by no means essential to produce the secondary effects of phlebitis and a fatal issue. For instance, after venesection pus may be secreted into the cavity of the vein, but hermetically sealed there by effusion of lymph agglutinating the sides of the vessel above and below. We remember witnessing a case of this kind at St. George's Hospital, where matter in the cephalic vein was bounded by an impassable barrier of lymph towards the upper extremity of the vessel, and yet the patient died from sero-purulent effusion into the cavity of the pleura. This fact also proves that Mr. Hunter's proposal to arrest the march of phlebitis by producing the "adhesive inflammation" between the wound and the heart, is an idle notion engendered by a subtle theorizing spirit. In fact, the cases of White and Crute (10 and 13) are *fatal* specimens of the "adhesive inflammation." We believe it must be conceded that we do not at all understand the modus operandi of phlebitis on the system, and that though the constitution of the fluids would seem in many instances to be deeply deteriorated, it would be unsafe and unphilosophical to argue on such deterioration in others. We must pass to another point.

"Before quitting the subject of the local affection, I would allude briefly to a circumstance which has escaped the notice of those who have previously treated of inflammation of veins, viz. the point at which the inflammatory changes in the coats usually terminate. According to my observation, these changes are limited by the passage of a current of blood; where a trunk is concerned, the boundary being the entrance of a branch, and where a branch is concerned, the boundary being the junction of this with the trunk." 47.

Our author's attention was first drawn to this point on examining the jugular vein of a horse which had inflamed after venesection, as well as by two other cases which he briefly mentions. In all, the inflammatory appearances ceased abruptly at the point of junction with the main trunk or the entrance of a considerable branch.

"I believe that in the case of Brancher, the inflammation in the basilick vein was bounded by the entrance of the subscapulary, the axillary vein not being affected. It will be found in the report of Carr's case, that immediately above the termination of the diseased appearances, the cephalick and several smaller vessels entered. In the instance of Goldinger, the inflammation extended in the cephalick, to where it terminates in the axillary. In the case of Mitchell, the morbid appearances extended in the common iliac, to where it joins the cava, which was not inflamed. In the case of Mr. A. related by Dr. Duncan, they spread in the cephalick, to its termination in the subclavian, which remained quite healthy.

"Of three cases of inflammation of the cava after parturition, seen by Mr. Wilson, in two the diseased changes ceased at the entrance of the venæ cavæ hepaticæ, and in the other, at the entrance of the emulgent veins. In the instance of Crute, it is distinctly stated that the deposition of lymph in the cava terminated at the entrance of the emulgent. And it would be easy to show, by referring to the details of several of the other cases, that the point at which the inflammation in the vessel is stated to have ceased abruptly, corresponds precisely with that at which certain veins enter." 51.

We confess that we think Mr. Arnott has attached an unnecessary importance to the foregoing point. It is obvious that unless the inflammation of the vein extend as Hunter erroneously supposed, to the heart, it must cease more or less abruptly at some point between the injured part and the cardiac extremity of the venous system. Now when we take into consideration the constant junction of branches with the greater trunks from the very extremity of the system to the cavæ, and even with them till they *débouchent* in the right auricle, we shall cease to wonder at the occasional cessation of inflammation at one of these points of junction. We say *oc-*

*casional*, for Mr. Arnott's own cases are sufficient to prove in the teeth of his conclusions that it is nothing more. In Case 5 the brachial, axillary, subclavian, external and internal jugulars were all affected;—in the case of Clementine, the 8th on the list, the inflammatory appearances extended from the median basilic *up into the subclavian opposite the scaleni;*—in the case of Thomas Fuller, the 10th, the median cephalic had been wounded and the inflammation passed up into the cephalic were it was lost *two inches and a half below the axillary*, consequently where no branch could have entered it;—in Thomas White, case 11, the "adhesive inflammation" extended *from the femoral vein to the cava, which was also inflamed though without lymph or pus;*—in James Boyle, case 12, the crural vein having been wounded was lined with pus and lymph *from the ham to the common iliac, and the disease extended for a considerable distance down the saphena;*—in John Crute, case 13, lymph was deposited *from the femoral vein to the emulgents;*—in case 15, Jane Strangemore's, purulent matter, lymph, and blood were observed *from the femoral vein to the cava beyond the diaphragm.*

Really we cannot conceive how Mr. Arnott could overlook these stumbling blocks to his position, which contradicted him, we may almost say, to his face. Even in the cases which he cites more particularly himself, circumstances will be found which shake his statement to its very base, circumstances which he does not and cannot explain. In Mitchell for instance, the 14th, the vein was inflamed from the face of the stump as high as the junction of the common iliacs to form the vena cava. Now why should the inflammation pass the entrance of the internal into the external iliac, if there were any real virtue in these venous coalitions? Phlebitis would seem to be a very capricious sort of inflammation, since it leaps in derision over one branch to stop at the rubicon-like influence of another! Even had not Mr. Arnott supplied us with the means of confuting himself in the cases to which we have referred, we should look with suspicion on such a pathological law as this, because it seems opposed to reason and analogy. We can readily understand how a deposition of lymph may bound an inflammation, and prove a *cordon sanitaire* in arresting disorganization. But how the entrance of one vein into another, and the presence of a fresh current of blood favouring by its direction the propagation of inflammation along a continuous tunic, can prove a *barrier* to that inflammation, we do profess ourselves unable to conceive. Fortunately *facts* step in to cut the knot, and save our wits from its bewildering difficulties. Facts, we say, are opposed to Mr. Arnott's supposition and the observations of future observers will only be offerings to its shades, its *dis manibus!* It was but a week or two ago that we witnessed a case at St. George's Hospital, in which phlebitis of the femoral vein succeeded amputation of the thigh. On dissection pus and lymph were found to be contained in the femoral and external iliac, but the inflammatory appearances were *not* arrested abruptly at the junction of the internal iliac or the cava. This fact was particularly noticed by several gentlemen in the hospital dead-house, in reference to the unsoundness of Mr. Arnott's views on the subject. In justice to Mr. A. we shall quote his concluding passage:—

"I have stated the foregoing facts regarding the limits of the diseased changes in Phlebitis, without pretending to explain how it happens that this same inflammation which has stopt short at the entrance or passage of a current of blood, may not only already have passed several currents, but have extended itself into the vessels conveying them." 51.

How significant a commentary this short but pithy acknowledgment of difficulty offers on our text;—

"A thousand years scarce serve to form a state;
"An hour may lay it in the dust!"

### SECONDARY AFFECTION IN PHLEBITIS.

"The secondary affection in Phlebitis, usually shows itself in from two to ten or twelve days after the receipt of the injury which has occasioned the inflammation in the vein; when the vessel has been previously diseased, sometimes sooner. The symptoms may be thus briefly characterised.—

"Great restlessness and anxiety, prostration of strength and depression of spirits, sense of weight at the precordia, frequent sighing or rather moaning, with paroxysms of oppressed and hurried breathing, the patient at the same time being unable to refer his sufferings to any specific source. The common symptoms of fever are present, the pulse is rapid, reaching sometimes to 130 or 140 in a minute, but is in other respects extremely variable. There is often sickness and violent vomiting, especially of bilious matter. Frequent and severe rigors almost invariably occur, sometimes to the number of three or four in the course of a few hours. The general irritability and deep anxiety of countenance increase, the manner is quick, and the look occasionally wild and distracted — When left to himself, the patient is apt to mutter incoherently, but on being directly addressed, is found clear and collected. The features are pinched, and the skin of the whole body becomes of a sallow, or even yellow colour.

"Under symptoms of increasing debility, and at a time when the local affection may appear to be in a great degree subsiding, secondary inflammations of violent character, and quickly terminating in effusion of pus or lymph, very frequently take place in situations remote from the original injury; the cellular substance, the joints, and the eye have been affected, but it is more particularly under a rapidly developed attack of inflammation of the viscera of the chest, that the fatal issue usually occurs. Whether this is observed or not, death is always preceded by symptoms of extreme exhaustion, such as those of a rapid feeble pulse, dry, brown, or black tongue, teeth and lips covered with sordes, haggard countenance, low delirium, &c." 53.

The duration of this affection varies considerably and that under circumstances that do not readily admit of explanation. In Mr. Hodgson's case, the 17th, the patient died four days after the division of the saphena, whilst in case 5, after venesection the patient survived till the end of the seventh week. The morbid appearances found in the bodies of those who have died of phlebitis are very remarkable, and are thus arranged with respect to their various situations by our author.

"In the Chest—effusions of sero-purulent fluid into the cavities of the pleuræ and pericardium, exudation of coagulable lymph on the surfaces of the heart and lungs, hepatisation of the latter organ, the infiltration of pus into its tissue,*

* "Pus was deposited in the muscular substance of the heart in a case of Phlebitis after venesection, which occurred subsequently to this paper being read to the Society, and an account of which, presented to me by Mr. Lawrence, will be found in the Appendix."

or small collections like a mixture of pus and lymph.* Such appearances presented themselves in ten cases (Carr, Goldinger, Mr. Broughton's case, Johnson, Dogherty, Clementine, Capt. L., Fuller, White, and Dodging,) out of seventeen; in three, (Boyle, Mitchell, and Mr. Hodgson's case,) the thorax was not examined; in two, (Crute and Strangemore,) the condition of its contents is not noted; and in two, (Brancher and Arnold,) no diseased appearances were observed.

"In the Cellular Substance, intermuscular as well as subcutaneous—pus and sero-purulent fluid has been extensively deposited, sometimes in collections like abscesses, at others, appearing more like an effusion into its cells, than as resulting from the common process of inflammation. These collections most frequently occur in the vicinity of the joints. In two cases (Brancher and Dogherty) pus was deposited under the skin of the opposite fore-arm, near the wrist; in one, (Arnold,) with inflammation of the knee-joint, into the intermuscular cellular substance of the corresponding thigh, and into that external to the joint of the opposite shoulder; in one, (Dodging,) into the intermuscular cellular substance of the opposite leg and of both fore-arms; in one, (Goldinger,) into the interfibrillar cellular tissue of the corresponding pectoral muscle, and in another, (Dogherty,) between the sternal extremities of the two first ribs and pleura.

"A Disease of the Joints,—consisting of a most violent inflammation of the synovial membrane, its distension with purulent matter, destruction of the cartilage, and baring of the bones (Arnold's case.) These changes too, taking place in the brief space of a few days, the knee having been first attacked with pain four days before death, which again took place in sixteen from the date of the injury of the vein which caused its inflammation. In two other cases, (Brancher and Dogherty,) there was affection of the knee-joint, but the parts were not examined after death.†

"In the Eye—opacity of the cornea, injection of its blood-vessels, and destructive changes in its humours or coats, occurred in Dodging.

"Besides these affections, there were found in five instances, (Carr, Arnold, Goldinger, Mr. Broughton's case, and Dodging,) within the cranium, opacity and thickening of the tunica arachnoides, effusion between it and the pia mater, and increased secretion into the ventricles. In nine, (Brancher, Johnson, Capt. L., Fuller, White, Crute, Boyle, Mitchell, and Mr. Hodgson's case,) the head was not examined; in three, (Dogherty, Clementine, and Strangemore,) no morbid appearances were noticed." 57.

We have few remarks to make on the foregoing summary, but we cannot refrain from commenting on the state of the lungs and liver in the cases detailed by Mr. Arnott. In *none* of these nineteen cases, including that of the horse, was any thing like a depôt discovered in the liver, and in only *five* was there aught approaching to an abscess or depôt in the lungs. Of these five two are extremely unsatisfactory, it being mentioned in one that there were *vomicæ* in the lungs, extremely different in every respect from the depôts, and in the other that there were "hepatized portions of lung

---

* "In the Appendix will likewise be found the history of a case of inflammation of the jugular vein in the horse by Dr. John Sims, where depositions of a similar nature had taken place into the lungs."

† "An instance of an affection of the knee and shoulder joints consequent to Phlebitis after venesection, equally destructive in its effects as in Arnold's case, has since occurred in St. George's Hospital, where, through the kindness of Mr. Keate, I had an opportunity of seeing the patient during life, and being present at the examination of the body after death. The case has been reported, Med. Gazette, Vol. II. p. 730."

gorged with fluid which was purulent in some." When Mr. Arnott tells us by and bye that the purulent depôts in the liver and the lungs depend upon *phlebitis*, it will be well to remember that out of nineteen picked cases of that kind, *none shewed any affection of the liver and only three a satisfactory implication of the lungs.** With respect to the opacity of the arachnoid membrane and the serous effusion between it and the pia mater we must also beg leave to observe that little dependence should be placed upon them as a test or consequence of this or that disease. Nothing is more frequent on opening dead bodies than such an appearance, and that in cases of diametrically opposite descriptions, where the patient has been delirious before death, and where he has not. Our readers will find some observations on this subject and cases in point in the 21st number of this Journal, p. 187, et seq. Since that time many additional instances of the kind have occurred under circumstances so various, that as we said before, we attach slight importance to the appearance in question.

Having remarked that although one or other secondary consequence of phlebitis is very common the disease occasionally proves fatal without them, a remark with which we perfectly concur, Mr. Arnott proceeds to compare the progress of the secondary affection to that of diseases arising from the inoculation of a morbid poison. Mr. Arnott particularly alludes to the sequelæ of dissection wounds, and points out in individual cases the disposition to purulent formations in distant parts of the cellular membrane, similar to what has been shewn to take place after inflammation of the veins. An allusion to this coincidence is all we can afford, and we accordingly pass on to—

### PART. II.

After adverting to the opinions of Mr. Cheston, Mr. Hunter, Morgagni, Monteggia, Mr. Guthrie, Mr. Bell, M. Velpeau, and Mr. Rose, on the purulent depositions in the internal viscera and cellular membrane that too often follow operations and wounds, Mr. Arnott puts forward his own theory in the following terms.

"In the statement which has been now made, I have not alluded to the theory which attributes the formation of these abscesses to a disturbance of the nervous system, the opinion itself being so purely conjectural, and the operation of the cause so undefined and unintelligible as to render this unnecessary. In fact, the only view of the subject supported either by evidence, or argument, is that which considers the origin of abscesses and inflammations in remote situations after injuries, as connected with the absorption into the circulation of purulent matter from a wound. That they do depend on the entrance of such fluid into the blood,

---

* We had ourselves taken notice of the tendency to sero-purulent effusion in the pleura as a consequence of phlebitis, long ere the present paper of Mr. Arnott. Most of our remarks on the subject are scattered in critical articles throughout the Journal, but if the reader will be kind enough to refer to the 18th No. for July, 1828, p. 474, he will perceive that we have noticed the fact very explicitly. At that time indeed we believed that the purulent depôts in the *substance* of the lungs were a common consequence of phlebitis. Subsequent experience, and the observation of many cases, have convinced us that we were in error in that respect. The observations attributed at p. 477 of the same No. to another, were in point of fact our own.—*Rev.*

the consequences which have been observed to follow Phlebitis simply, sufficiently testify, and it becomes a question, whether the occurrence of Phlebitis, and the passage of pus from an inflamed vein into the circulation, is not of itself sufficient to account for the secondary affections of wounds, without its being necessary to resort to an absorption of the same fluid from their suppurating surfaces.

"The secondary affections succeeding to wounds, are, effusions of pus and sero-purulent fluid into the cavity of the chest, and inflammation of the pleura; similar affections of the cellular substance; effusion of pus into, and inflammation of the synovial membranes; depositions of pus and tuberculous abscesses in different organs of the body, viz. in the brain lungs, heart, liver, spleen and kidney.

"Now when it is considered that abscesses have formed in various parts of the body from the ligature merely of a vein, as of the saphena*—that pus was deposited under the skin of the fore-arm in the case of Brancher,—that rapidly destructive inflammation in the knee-joint took place in the case of Arnold,—that the same occurred in the eye in the case of Dodging,—that where symptoms of inflammation of the chest were observed during life, the effects witnessed, on examination after death, were of very disproportionate degree and extent,—and that effusions of coagulable lymph, and sero-purulent fluid into the chest, together with abscesses in the lungs, were found where no symptoms had indicated their existence;—I say, when it is considered that *all* these consequences ensued from so simple and definite an injury as the puncture, division, or ligature of a vein, it is impossible to resist the supposition that, where similar secondary affections have succeeded to a more extensive wound, they may in reality have originated in the same cause, viz. inflammation of a vein or veins.

"If such view of the subject is correct, we ought on the one hand, in cases where the consequences already mentioned have succeeded to wounds and injuries, whether of the extremities or head, to find evidences of inflammation of the veins of the part which had been primarily or mechanically injured, and, on the other, to meet with similar secondary affections in cases where inflammation of the veins is known to be of common occurrence, as after parturition; that this is actually the case, I shall now proceed to shew." 69.

If Mr. Arnott imagines that he can *put out* the theory of the agency of the nervous system with a paragraph he will find himself wofully mistaken. So we are to abandon the notion of nervous agency because forsooth it is "undefined and unintelligible." It is cutting the knot to be sure to destroy at one fell swoop the powers of that system which connects us with the external world, which is the source of our pleasures and our pains, and does more to produce and to modify disease than each and all of the other systems of vessels or of parts that make up the organic machine. Is man distinguished from other animals by the superior arrangement of his bloodvessels, his muscles, or his bones? Certainly not, for many of the inferior creatures shew contrivances as wonderful and arrangements as perfect as the lord of the creation. No, it is in the complexity and the perfection of his nervous system that man stands pre-eminent: it is in that, and in that almost alone. And yet there is a kind of school in modern times who would throw this system overboard in the consideration of disease, who because they cannot demonstrate its agency deny it, and would seem to consider the brain, the cerebral and

* "Carmichael in Trans. of the King and Queen's College of Physicians. Vol. II. p. 346."

spinal nerves, and the intricate ganglionic system as leather and prunella, as a bug-bear for grown children to be laughed at by their philosophy!

*Quis furor iste novus ; quo nunc, quo tenditis ?*
*Heu miseri cives !*

They refuse all belief to, they treat with contempt the operation of the nervous system because it is "undefined and unintelligible." When a man receives a blow at his scrobiculus cordis and dies as if struck by a cannon-ball, we say that it is through the medium of an impression on the nervous system, and yet we can neither define nor understand its operation. A piece of bad news has produced the jaundice in a few short minutes, a deep impression of grief or terror has turned the hair of a young man grey in a single night, but would Mr. Arnott dispute in such cases the agency of the nervous system, albeit that neither he nor any man can define or understand it? He could not, he would not be so bold. We fear it is the mania for morbid anatomy which is operating injuriously by blinding men's eyes to every action in the living body that is not capable of mathematical demonstration. We have lately got so much into the habit of *bottling* disease, that we fancy no morbid process can go on, which we are unable to shew in spirits on the shelves of a museum.

To revert to the operation of the nervous system in the present description of cases more particularly, we believe that it is not so contemptible as Mr. Arnott would wish us to imagine. If a patient after a slight operation become ssuddenly affected with an insidious visceral inflammation and dies, and if upon dissection the original wound is found nearly healed and no other tangible cause for the secondary phlegmasia is discovered, must we not refer its occurrence to some more occult and inexplicable agency? We may slight the nervous system certainly, but we only pass from the mud to the mire, and abandoning a feasible solution of a difficulty obtain none at all. Take for instance the following case which we witnessed at St. George's Hospital.

*Case* A.* A young man, four or five and twenty, had part of a prepuce thickened after paraphymosis removed by Mr. Keate on the 20th of Feb. 1829. Next day the prepuce was swollen and the patient feverish; on the 22d the local uneasiness was relieved but the patient was unusually nervous and irritable; on the 23d he was in a state of extreme prostration and at noon of the 24th he died. He had not been affected with cough or the slightest dyspnœa throughout.

*Sectio Cadaveris.* Nothing particular about the penis—wound nearly healed—no suppuration nor any thing like it, and no tumefaction of the prepuce. Pleuræ on right side of thorax bound together by old and recent adhesions—middle and inferior lobes of right lung united by a flake of strong, recent, yellow lymph, half verging into pus—inflammatory *engouement*, and in some places almost hepatization of the lung. A few flocculi of recent lymph between the pleuræ on the left side—this lung very extensively hepatized and impervious to air from recent pneumonia. Arachnoid

* We have marked the cases brought forward by ourselves with the capital letters A—B, &c. to distinguish them from those of Mr. Arnott.—REV.

membrane of brain opaque with serum effused between it and the pia mater —some fluid in the ventricles.

We leave Mr. Arnott to explain the quomodo of the pleurisy and inflammation of the lungs in the foregoing case, in which it was evident that there was no phlebitis. In the following the evidence is also very strong against him.

*Case* B. — Childs, æt. 66, of a bad habit of body, had the right breast amputated for scirrhus at St. George's Hospital on the 26th Feb. by Mr. Brodie. A very slight secondary hæmorrhage occurred but required no removal of the dressings, and from the 27th of February to the 2d of March she appeared to be doing very well. On the latter day she had a rigor, followed by sickness and fever, and on the 3d an erysipelatous blush appeared about the right breast and shoulder; breathing rather quick—expression anxious—wound looking healthy. On the 4th she felt chilly now and then and had a little pain in the right side on inspiration—wound healthy. In the night she complained of pain in the left lower extremity, which on the 5th presented diffuse swelling, tenderness, and a glossy state of skin, with a slight patch of redness near the inner malleolus; pulse rapid—delirium—Hippocratic countenance. On the 6th the whole extremity was mottled, cold, and in a state of gangrene; the wound was dry and its lips gaped. About 2 p.m. she died, eight days after the operation.

*Sectio Cadaveris.* Nothing particular about the wound—a couple of drachms of pus in the pectoralis major in the neighbourhood. Leg in a state of mortification with vesications, &c.—muscles and cellular membrane sloughy and semi-putrid—a purulent depôt in the cellular membrane behind the malleolus internus—pus in the ankle-joint without ulceration of its cartilages—thoracic and abdominal viscera sound—head not examined. The femoral and tibial vessels, both artery and vein, were carefully examined by Mr. Brodie *and found to be perfectly sound.*

This case does not square with Mr. Arnott's doctrines, but facts are facts. We shall now resume our analysis of the memoir.

### Affections of Viscera, Joints and Cellular Substance after Injuries of the Extremities.

In the four following cases depositions of the above kind were connected with unequivocal phlebitis.

*Case* 1. Under Mr. Arnott's own observation.

J. R. æt. 52, had the left leg amputated on the 18th November, 1826, on account of mortification of the foot succeeding to a severe bruise from the tread of a horse. Two veins were necessarily tied, the wound did not unite, much constitutional disturbance with great depression succeeded, and death took place on the 3d of December. *On Dissection:*—much sero-purulent fluid in the left cavity of the thorax, and coagulable lymph on the pleura pulmonalis—a small abscess in the right lung—capsule of the left hip-joint distended with purulent matter, and its cartilages ulcerated—similar appearances in the opposite hip-joint—coats of the saphena vein thickened and its cavity for the last two or three inches of its course filled with pus unconfined by any lymph.

*Case* 2.* A man with compound fracture of the left leg, was attacked after a time by fever, rigors, and a yellow colour of the skin. He died within a month. *On dissection:*—four abscesses in the right lung, several in the left with sero-purulent fluid in the pleural cavity. Crural vein inflamed and its cavity filled with purulent matter; the inflammation extending as high as the cava.

*Case* 3.† Henrius, æt. 19, had the second metatarsal bone of the right foot amputated. On the 9th day the skin became jaundiced and a purulent deposit formed on the back of the left hand; on the evening of the 10th he died in great exhaustion. *On dissection:*—a number of small abscesses in the left lung—infiltration of the cellular substance of the left fore-arm and arm with pus—an ounce and a half of thin yellow pus in the left shoulder-joint without inflammation of the synovial membrane—veins on the dorsum of the foot red, thickened, and filled with pus.

*Case* 4.‡ Frederick Wells, a robust countryman, æt. 25, had the right leg amputated for compound fracture of the metatarsal bones on the 9th of June, four hours after the accident. The stump became sloughy, the complexion sallow, he had rigors, pain was felt in the course of the vessels and he died nine days after the accident. *On dissection:*—several purulent depôts in right lung—liver large and ecchymosed upon its surface—femoral vein filled with pus from the ham to the point where it is joined by the profunda, and its inner surface coated with thick lymph.

"Sero-purulent effusion into the chest—abscess in the lungs,—disease of the joints and cellular substance having been found to succeed to Phlebitis simply, may not the same occurrences in these four cases be referred to the inflammation and suppuration of the veins which existed?" 73.

It might or it might not, but supposing that cases are brought forward in which these visceral depôts were to all appearance unaccompanied with phlebitis, would they not shake the doctrine as one of universal application? But we do not consider it as proved beyond dispute that inflammation of the veins was the cause of the depositions, even in the four cases cited by Mr. Arnott, as strong ones too as any that he could select.

In the 4th case, for example, the patient died on the ninth day after the operation with phlebitis and purulent deposites in the lung. Now the deposites if a consequence of phlebitis at all are a *secondary* consequence, occurring after the phlebitis itself has existed a certain length of time. In Case 5 of Mr. Arnott's paper, when unequivocal inflammation of the vein after venesection was followed by the formation of small abscesses in the lungs, the patient did not die till the end of the seventh week. In a case which we witnessed at St. George's Hospital, where phlebitis after venesection was followed by destructive suppuration in the right shoulder and knee-joints, the patient lived upwards of six weeks:—in fact, if the reader will glance at the eighteen cases of phlebitis succeeded by suppuration in various parts, detailed in the first division of the present memoir, he cannot

* Palletta, Exercitationes Pathol. Milano, 1820, p. 21.
† Velpeau, Revue Médicale, Juillet, 1826, p 68.
‡ Lond. Med. Gaz. Vol. II. p. 127.

fail to observe that in most of them a certain space of time elapsed before the development and fatal termination of the secondary suppurations in question. The bearing of these statements on the present subject must be obvious, for if a patient dies on the *ninth or tenth day* after amputation with phlebitis *and* purulent deposites in the viscera, they would lead us to hesitate before we yielded implicit assent to the opinion that the latter depended exclusively *on* the former. The same cause, an operation or injury, which produced suppuration in a vein might also produce simultaneous or nearly simultaneous purulent infiltrations elsewhere.

But Mr. Arnott will probably deny, as indeed he does by implication in more than one place, that such visceral deposites take place at all independent of phlebitis. Setting aside the cases of *Carey* and *Childs* which we have already related, we shall endeavour to prove by the mention of other cases that occurred under our own observation that the connexion of the two diseases is not of the intimate nature our author would lead us to imagine. We shall first mention two instances of phlebitis without depôts and then some of depôts without phlebitis.

*Case* C. David Lawrence, æt. 56, admitted into St. George's Hospital October 23d, 1827, under Mr. Brodie, for a varicose state of the veins of the right leg and foot, and a small ulcer near the inner ankle. He said that some years previously a cluster of small veins over the head of the tibia had inflamed, become solid, and subsequently obliterated or nearly so;—at the time of his admission no trace of them remained. On the 1st November one of the largest veins winding round the inside of the thigh to the saphena was found to have become partially filled with coagulum, and appeared to be thickened in its coats; it was rather tender on pressure. He went on well till 6, a. m. of the 15th, when he was seized with a sharp rigor; he had malaise during the day, and suffered in the night from pain in the obstructed vein. Next morning inflammation of the saphena, with swelling of the thigh, &c. was established, he became incoherent, and on the 17th gangrene of the limb, without vesications, ensued. At 2, p. m. he died.

*Sectio Cadaveris.* Cellular membrane of the thigh loaded with serum—coats of the inflamed veins much thickened, and their cavities filled with loose broken down coagula—internal venous tunic from the groin to the heart stained of a modena colour by the blood which was universally fluid—heart itself flabby—inner coat of the aorta and its branches stained like that of the veins, but of a brighter tint. *Nothing unusual found in any of the great cavities.*

*Case* D. Martin Griffin, æt. 35, admitted into St. George's Hospital January 28th, 1829, under the care of Mr. Brodie. *Right* lower extremity generally swollen, pitting on firm pressure, increased in temperature and painful at nights—extreme tenderness in the course of the saphena major vein, especially in the thigh, where its coats were thickened and felt like a hard cord beneath the integuments—other cutaneous veins very full and tortuous—health not much affected. Five months before admission had a severe bowel complaint from which he recovered, but exposure to wet and cold produced a relapse with rigors, sickness, and fever. The *left* leg and thigh then swelled and presented precisely the same state of things as the right do now; under the use of leeches, &c. the limb resumed its natural condition in the course of two or three months. The affection of the *right* lower extremity is only of three weeks' duration, appeared without any assignable cause, and was ushered in by fever. We need not detail the treatment had recourse to, suffice it to say that by the 11th of February the hardness and thickening in the course of the saphena had nearly disappeared and the swelling of the extremity was gone.

Here we see two indisputable cases of phlebitis presenting some points of analogy with each other, but neither evincing the slightest disposition to deposites in distant parts. We shall now reverse the order of phenomena and relate some cases where the latter were discovered without any appreciable inflammation of veins.

*Case* E. *Depôts in the Lungs after Amputation.* John Cooper, æt. 14, of scrofulous aspect, from the country, was admitted April 9th, 1828, under the care of Mr. Brodie, with necrosis and abscess of the left tibia. On the 24th amputation was performed above the knee, and a good many vessels required to be tied. The pulse after the operation continued high, and the stump put on a sloughy appearance. On the 4th of May there was a disposition to vomiting and pain on pressure of the abdomen or thigh; on the 6th he was attacked with purging, and next day began to be delirious; in the evening of the 11th he died. The quickness of the pulse and excessive irritability throughout were remarkable; he had no rigor as far as could be ascertained.

*Sectio Cadaveris.* Inflammation of pleuræ on right side, layer of recent lymph, about an ounce and a half of sero-purulent fluid—no fluid on left side but preternatural vascularity of the pleuræ, especially in spots corresponding to superficial deposites of pus, some as large as hazel nuts interspersed over the surface of both lungs. Liver rather large but healthy—mesenteric glands rather large. Stump sloughy and the bone protruding—quantity of dark coloured fetid pus burrowing in the cellular membrane between the origins of the adductors longus and magnus—periosteum in parts detached from the bone by pus insinuated between them, and in parts actually sloughing—*blood-vessels slit open and found to be free from inflammation and to contain no pus.*

*Case* F. *Depôts in the Lungs after Compound Fracture.* Henry Rose, æt. 25, a drayman, admitted October 1st, 1828, under the care of Mr. Brodie with comminuted compound fracture of the left leg into the ankle joint. Diffuse inflammation of the cellular tissue with a dark brown tint of skin supervened, the cellular membrane sloughed extensively, and free scarifications or rather incisions were employed with much benefit to the limb. On the 7th he was hurried in his answers; on the 8th the conjunctivæ were muddy and the skin beginning to be yellow; on the 9th the bilious tint was distinct and he complained of pain in the right side of the chest and hypochondrium increased on inspiration, coughing, or pressure; on the 10th the discharge from the limb was more scanty; on the 11th he had several rigors; in two or three days from this period he died.

*Sectio Cadaveris.* Cellular texture of leg beneath the skin and between the muscles in a horrible sloughing disorganized condition—on the outer and inner side of the thigh it was better, but on the posterior part it was in a very bad state—*femoral vessels perfectly healthy*—traces of recent pleurisy in both sides of the chest—one small depôt in the upper part of the right lung—several large and sloughy ones at the lower margin of the left.

*Case G. Serum and Lymph in the Pleura and Pus in the Knee-joint after Erysipelas.* Joseph Smith, æt. 53, admitted Aug. 27th, 1828, under the care of Mr. Brodie with extensive varicose ulcers of the right leg and thigh. On removing the strappings which were used, on the 5th Sept. the limb was found to be the seat of severe erysipelas at that time prevailing in the house. The tint of the erysipelas was dusky, the depression of the system considerable. On the 8th the erysipelas was fading in some degree, but the skin had a yellow tinge, the manner hurried, no pain on a full inspiration; on the 9th the erysipelas had spread up the right side of the body and was complicated with vesications, the countenance of decided bilious hue. He died in the course of the evening.

*Sectio Cadaveris.* Textures of the limb not gangrenous, but the surface somewhat purple coloured—right knee-joint found filled with pus, its cartilages ulcerated, and its synovial membrane thickened—femoral vessels healthy but stained as was the aorta—serum and lymph in either cavity of the pleura especially of the right with pus on the pleura pulmonalis.

We think these three cases conclusive in proving that purulent depositions may take place independent of any inflammation of the larger veins. We might multiply instances of the same kind after compound fracture in cases that have fallen under our own observation, but such a proceeding would merely tire the patience of our readers without affecting the question at issue. We shall presently discuss the theory, for such it is, that inflammation of the *smaller* branches of the venous system is sufficient to produce these secondary depositions, and in the mean time pass on to—

### AFFECTIONS OF THE VISCERA, &c. AFTER INJURIES OF THE HEAD.

"Abscesses of the liver after injuries of the head long excited much curiosity, and a variety of speculations were indulged in, to account for the occurrence. They were attributed by some to an increased quantity of blood returning from the head through the superior vena cava, proving an obstacle to the ascent of that through the inferior; by others, to a smaller quantity of blood being carried to the brain, and consequently a larger quantity being sent to the abdomen; they were attributed by some to concussion of the brain, and the nervous system was supposed to be the principal agent in their production; by others, they were referred to direct injury of the liver at the time of the accident. The first two of these speculations fell to the ground, when it was remarked, that abscesses occur in the viscera of the chest, as well as in those of the abdomen, after injuries of the head, *and the two last, when it was observed, that they have taken place when neither the brain nor the liver have been the subject of concussion or of direct injury.*

"But of what nature are the injuries of the head, to which secondary affections of the viscera, of the abdomen, and chest succeed, and what are the circumstances under which these occur? Upon neither point do systematic writers afford us any information; and those whose attention has been more particularly directed to the subject have been too much occupied with their own hypotheses, and their own limited observation, to allow them to form a comprehensive and correct estimate of these circumstances.

"With the view of supplying this deficiency, and of enabling us to arrive at more satisfactory conclusions as to the origin and cause of secondary affections in distant parts after injuries of the head, I have referred to a number of cases, and, taking every one which presented itself, I have noted the following particulars." 74.

We cannot forbear remarking with how impotent a blow the doctrine of nervous agency is supposed to be overthrown, and how premature the conclusion that *because* visceral abscesses take place independent of any direct injury to the encephalon, they must necessarily be also independent of its influence. It is *pure* surgery in perfection to imagine that the mysterious agency of the nervous system is only aroused by a blow or a bludgeon! The brief but sufficiently complete details of thirty-three cases of the secondary consequences of injuries of the head are furnished by Mr. Arnott. For these we must refer to the memoir as there is no necessity for our transcribing them here.

"From a consideration of these cases, it appears that affections occurring as secondary to injuries of the head were observed, in twenty-one, seated in the abdominal viscera;* in five, in the thoracic; and in six, in the abdominal and thoracic conjointly. That they consisted of collections of pus in the liver and in the lungs; and of effusions of pus and sero-purulent fluid into the cavities of the chest. That combined with some of these, there was further observed, a deposition of purulent matter, in one case, in the substance of the heart; in one, in the kidney; in one in the spleen; and in one, under the integuments of the back; in one, albuminous effusion on the surface of the intestines; and in one, inflammation of the liver, without the formation of matter. In two cases, inflammation of the surface of the liver without suppuration was the only morbid appearance observed. In one case, an affection of the joints, and the deposition of pus into the cellular substance around them occurred without any disease of the abdominal or thoracic viscera having been noted.

"The injury which the head had sustained in these cases consisted, in twenty-three, of fracture or fissure of the cranium, in all compound, with the exception perhaps of two, where the circumstance is not stated. In ten, the osseous covering of the brain was neither fractured nor fissured; but with the wound of the soft parts, which uniformly existed, there had been in several a portion of the outer table and diploe sliced off, whilst in all, the bone seems to have been exposed. The wound of the soft parts was in several instances of trifling extent, and had, in some nearly, and in one actually, healed over when the unfavourable symptoms commenced; but in these cases disease of the bone seemed to have remained, as the pericranium now became detached. With regard to the morbid appearances within the cavity of the cranium, it will be sufficient to state, that whilst these vary considerably, in several there was inflammation simply of the dura mater, without any appearance of purulent matter, and that in three instances no diseased changes whatever were observed. In short, the only circumstance these thirty-three cases have in common is a wound of the soft parts.

"The general course of these cases seems to have been this, and in the great

---

* "Although these thirty-three cases show the nature of the secondary affections succeeding to injuries of the head, they cannot be considered as offering a fair representation of the comparative frequency of the occurrence in the abdominal and thoracic viscera. Previous to the time of Morgagni, abscess of the liver subsequent to such injuries had in a measure engrossed attention, and accordingly it will be seen that, out of thirteen cases from writers previous to his time, in twelve, disease of the liver is noted, and in two of these only, the condition of the thoracic viscera mentioned."

majority, twenty-four, it is so stated, that the patient for some time did well, having recovered his consciousness, where this had been lost, which was frequently not the case, was free from fever, and the wound suppurating kindly; that afterwards unfavourable symptoms took place, consisting of fever, rigors, nausea and vomiting, delirium, yellow colour of the skin, and sometimes, shortly before death, pain in the right hypochondrium, or affection of the chest. There was some difference in the period at which these symptoms appeared; but of nineteen cases, the earliest of which was the seventh, and the latest the twenty-fourth day, the average was between the thirteenth and fourteenth day after the accident. The average period of the fatal termination of the same cases was between the twenty-second and twenty-third days, the earliest being on the fifteenth, and the latest on the thirty-seventh subsequent to the injury. In one instance, not included in the above number, the patient did well until the eightieth day, when, on an attempt being made to remove a portion of bone adhering to the dura mater, general disturbance took place, and death in a few days. In another exception to the more ordinary periods, the same event occurred four months and a half after the receipt of the injury." 97.

The foregoing are strict deductions from the cases and unexceptionable in every respect; the following remarks are of a somewhat different cast:—

"From the preceding summary, I think it will appear, that abscesses and inflammations occurring in the viscera of the abdomen and chest, after injuries of the head, present a resemblance to similar affections succeeding to wounds of other parts of the body, sufficient to justify the inference, that they arise from the same cause. That cause, I ventured to suggest, might be inflammation of the veins,—the consequent production of pus in their cavities, and the entrance of this into the circulation. And, in accordance with this view, we find that in the only cases in which the state of the part is described, (number 21, from Schmucker, and number 32, which occurred in St. Thomas's Hospital) inflammation of the superior longitudinal sinus existed, its cavity in both instances, containing purulent matter, in the one, with a firm, fibrinous coagulum, and in the other, with a layer of organized lymph on its inner surface. But we need not confine ourselves to inflammation of the sinuses within the cranium in cases of this description. It must be evident, that this process taking place in the numerous veins which ramify between the two tables of the skull, and in those distributed to the soft parts externally, will be attended with similar consequences to those which succeed to phlebitis in other parts of the body.

"With respect to inflammation of the cerebral sinuses, after injuries of the head, I may here mention, that Schmucker* relates another case, where a patient having compound fracture of the os frontis, did well for ten days; unfavourable symptoms then occurred, and he died on the eighteenth, having for the last two days complained of pain in the right side. In this instance, a spiculum of the inner table of the skull was found penetrating the superior longitudinal sinus, which, in its anterior part, contained an atheromatous looking matter, and posteriorly, pus mixed with blood and polypous concretions. The condition of the abdominal and thoracic viscera is not described; but it is subsequently remarked, that the inflammation of the liver in this case would undoubtedly have ended in suppuration, had not death intervened." 98.

We cannot compliment Mr. Arnott on the logic or the fairness of the preceding observations, in fact we consider them as shewing unwittingly but broadly the cloven foot of theory. In only *two* cases out of 33 was inflammation observed in any of the sinuses of the head, *ergo* says Mr. A. inflam-

* "Chirurgische Wahrnehmungen. Erster Theil, p. 160.

mation of such veins or sinuses is the essential part of all! This reductio ad absurdum which the argument so palpably admits, seems to have occasioned some premonitory qualms in our author's mind, for he shortly abandons his position and takes up a new and extraordinary line of defence;—it must be evident, says he, that inflammation taking place *in the numerous veins which ramify between the two tables of the skull, and in those distributed to the soft parts externally*, will be attended with similar consequences to those which succeed to phlebitis in other parts of the body. Now this is evidently theory and nothing else, a theory too which cuts its own throat. In the first place Mr. Arnott assumes that there is inflammation of the smaller veins, and in the next place he assumes that inflammation of those small veins is attended with the same symptoms as that of the larger ones. The first supposition may or may not be the case, and the second is extremely unlikely. The question however had better be mooted something more in detail.

It must be evident, to use Mr. Arnott's hasty expression, that he has no more right à priori, to *suppose* inflammation of the smaller veins in such cases as injuries of the head, than we have to *suppose* it in cases like those of psoas abscess where immense destruction of parts is occasioned by the burrowing and collection of purulent matter. In vomicæ of the lungs too we are all aware how frequently bloodvessels even of considerable size and veins of course, amongst them, are implicated in the adhesive, suppurating, or sloughing actions going on in the pulmonary structure. In the enormous and ruinous sloughing abscesses that invade the subcutaneous and deep-seated cellular membrane of the thigh in individuals of strumous habit, abscesses by no means very chronic in their march, the smaller veins must inevitably become inflamed. Are purulent deposites then frequent in these cases of psoas abscess, vomicæ, &c.? By no means, indeed we never saw one instance of their occurrence under such circumstances. It would be unnecessary to pursue the subject any further as we think Mr. Arnott has failed to make out his case, and instead of advancing any sober or convincing facts, has put forward a mere assumption, unsupported by aught but a *brusque* and magisterial tone. The first part of the memoir affords a strong contrast to the present in this respect. In that Mr. Arnott brought forward a most valuable collection of cases of phlebitis and offered few opinions that were not pretty fairly deducible from them, but we still know too little of the proximate cause of the purulent deposites to justify an author in dogmatizing on their nature. We now pass on to—

Affections of the Viscera, Joints, Eye, Cellular Substance and Skin after Labour.

This is really a very rambling kind of chapter and a strange compound of facts and hypotheses, strict conclusions and bold assumptions. The author commences by citing Drs. Clark and Davis, M. Velpeau and others, to prove that inflammation of the uterine veins is by no means of unfrequent occurrence, and proceeds to observe that there is sufficient evidence to shew that it is followed by various secondary affections. In a case of Mr. Wilson's when inflammation had extended from the uterine and spermatic veins to the cava an abscess was found in the liver; in a case of M. Louis' there were abscesses in the right lung and inflammation of the right spermatic vein; in a case that occurred in the St. James's Infirmary under the care of Mr. Baker there were great effusion of turbid serum and lymph into the left side of the chest, and a portion of the lung as large as a walnut in a state of complete gangrene, with decided inflammation of the left spermatic vein, which was filled with lymph and pus; and finally, in a case lately received in the Middlesex Hospital under the care of Mr. Mayo, from the Queen's Lying-in Hospital, severe erysipelas supervened to labour, matter formed on the outside of the left fore-arm, back of the left hand and over the right wrist, whilst gangrene attacked the integuments of the right leg and foot. On dissection about a tea-spoonful of matter was found between the neck of the bladder and symphysis pubis, and pus and sero-purulent fluid were found in the lower half of the left spermatic vein with slight thickening and no lymph.

These are the four cases adduced by Mr. Arnott to demonstrate the tendency of uterine or spermatic phlebitis to give rise to secondary and remote suppurations. For our own parts we must say that they are extremely unsatisfactory and by no means establish the soundness of the conclusion so decisively as Mr. Arnott appears to imagine. The last case is assuredly not in his favour, for if it proves that inflammation of the lower half of one spermatic vein gave rise to any thing it was to *erysipelas*, an etiological bastard which we shrewdly suspect that Mr. Arnott will be in no hurry to have sworn to him!

The next series of affections which our author proceeds to consider are the affections of the joints which are known to supervene occasionally to parturition. After adverting to the fact of suppuration in the articulations being not unfrequently connected with phlebitis, as instanced in the cases of Arnold, Brancher, and Dogherty, and quoting Dr. Hennen, Mr. Rose and others to shew that it has been noticed after injuries and operations, all which is in our opinion undeniable, Mr. Arnott observes:—

"It will be inferred from the preceding course of argument,* that I regard the disease of the joints, in these several cases, as having been connected with inflammation and suppuration of the veins of the part which had been the seat of mechanical injury. To others, this opinion may seem scarcely warrantable by the degree of knowledge which is yet possessed upon the subject; so strong, however, is my own conviction of the reality of such connexion, that in the following case, where no injury had been sustained, the disease of the joints, and the occurrence of abscesses in different parts of the body, led me to anticipate the existence of inflammation and suppuration of the veins, which anticipation being confirmed by dissection, tends evidently to establish the accuracy of the previous conclusions. The subject of the case was a patient in the Middlesex Hospital, who attracted my attention soon after he had been transferred from the physician's ward to that of the surgeon." 109.

*Case.* T. Griffin, æt. 30, became feverish without assignable cause, was attacked with rheumatism in all his joints especially the left knee and right shoulder, and several abscesses formed in the right side of his neck. He entered the hospital and went out relieved in five weeks, but was exposed to wet and cold, had a relapse, and was five weeks in bed without advice. In Nov. 1827, six months from the first attack, he re-entered the hospital

* This is a misapplication of a term, as what went before was merely an enumeration of certain facts; *argument* is a suspicious word when applied to such.

under Mr. Bell, with great swelling of the left knee which was almost immoveably flexed, loss of power over the right arm, an abscess over the inferior angle of the scapula and another over the right sacro-iliac symphysis. The swelling of the knee-joint increased, an abscess formed over the bend of the fibula, and the head of the tibia became dislocated backwards, another abscess formed over the right shoulder and on the 13th Aug. 1828, the man died. *On Dissection*:—The cartilages and ligaments of the left knee and right shoulder-joints completely destroyed, and the heads of the bones surrounded by a quantity of thick purulent matter contained in a cyst formed by a blended mass of muscle and cellular substance—right hip-joint healthy —femoral vein twice its natural size, greatly thickened in its coats, and filled with lymph and pus slightly tinged blood. The same appearances were observed in the saphena, profunda, epigastric and smaller veins, and extended in the femoral from two inches below the profunda to the entrance of the internal iliac when they ceased; nothing unusual in the viscera.

"It is to be remarked, that this patient had never complained of the right thigh, or leg; it is true, he had been confined to his bed for the last eleven months, but retained the power of moving this extremity. Latterly, both this leg and the other had become œdematous. No cause could be discovered for the inflammation of the veins; the limb shewed no sign of external violence, with the exception of a small cicatrix on the inside of the leg." 113.

So much for the "prefatory remarks," the gist of which seems to be that affections of the joints have been noticed after injuries and operations, and that Mr. Arnott has seen a case where such affections were connected with inflammation of the femoral vein and its branches. He next proceeds to shew that violent diseases of the joints are also observed after parturition.

That a violent and destructive disease of the joints takes place in the puerperal state, although not attracting particular attention, the following details will show. Mr. Cheston, in his chapter 'On White Swelling,' treating of suppuration of a joint, its symptoms, and the danger which attends it, has the following note;—'A critical deposition in the joints is frequently productive of a similar event, (viz. suppuration,) and many women in particular, as Dr. Simpson has observed, have contracted it under the diary fever they are subject to in childbed. Of this a remarkable case has lately fallen under my care, where the patient was saved by a timely amputation.' Unfortunately the case is not related, and Dr. Simpson's remark is merely this, 'several of those I have had under these cases, could give no account of the rise of the trouble. Some women have contracted it under the diary fever (the weed) they are subject to in child-bed.'

"The following case detailed by M. Cruveilheir,* in a paper entitled 'Usure des Cartilages Articulaires,' will be found interesting, both from the period at which it occurred, and the nature of the affection.

"A lady, after a severe labour, was attacked by acute articular rheumatism, which successively affected all the joints. The pains diminished, but did not subside; by degrees, the knee-joints became stiff, and she heard or felt a crepitation on the least motion, even in turning in bed. The hip, the shoulder and elbow-joints, those of the wrist and metacarpus, soon became affected in a similar way. Various remedies were tried. Moxas were applied to one knee, and were imagined to afford relief; but at the end of three months, which time they took in healing, the joint was as stiff as before. M. C., who had latterly seen the patient recommended daily motion of the limb, but the pain which this occasioned could not be borne. It was hoped that anchylosis might put a stop to the sufferings, but this did not take place. At length, in endeavouring by ex-

* "Bibliotheque Médicale. Janvier 1827, p. 80."

amination of one of the joints of the fingers to ascertain the nature of this singular disease, a grating was recognized, and the same sensation being verified in all the affected joints, palliatives only were henceforth resorted to.

"On enquiring of Dr. Merriman concerning a disease of the joints in puerperal women, that gentleman informed me that he had seen such cases, but that he did not know of one which had terminated favourably.

"In the following case communicated to me by Dr. Robert Lee, no examination of the body was made; but the relation of it here, may, by calling attention to the disease, lead to an examination, in fatal cases, of the affected joints, and also into the state of the uterine veins, with inflammation of which, I believe the affection of the joints to be connected.

"'Mrs. A. æt. 30, was delivered on the 1st of June, 1828, after a tedious labour. A portion of the placenta having been retained in the uterus several hours after the birth of the child, a profuse hemorrhage took place before it was extracted. Until the 10th she appeared to recover in the most favourable manner, when a violent febrile attack was experienced, with delirium, and a painful diffused swelling soon after took place around the right knee-joint.

"On the 13th, when I first saw her subsequent to delivery, the febrile symptoms continued unabated, she was delirious, and there was a peculiar expression of wildness in the countenance. The muscles of the face and extremities were affected with tremors. The pulse was 130, and very weak; respiration hurried and anxious, with frequent cough. The skin hot and dry, the tongue was of a glossy red colour and moist. Thirst not urgent; bowels open. There was no sickness or vomiting, the abdomen was uniformly soft, and pressure over it produced no uneasiness. The right knee-joint was stiff and swollen, but the integuments were not discoloured. On the 14th, the symptoms continued, and in the night a painful circumscribed swelling had taken place in the middle of the calf of the right leg, where the integuments were hot, and of a dark red colour. On the 18th, there was a marked remission of all the symptoms, and for ten days it was hoped she would recover. From the 1st of July till the 24th, when she died, (completely worn out with diarrhœa, fever, and the painful affection of the extremities,) the right knee-joint had become much more swollen, and a considerable effusion had taken place into it. Over the right radius and ulna, near the wrist, a painful diffused swelling also took place without discolouration of the integuments, and for a week she suffered excruciating pain in the left ankle and right shoulder joint, but in neither of these situations, was any thing, except a slight puffiness to be perceived.'" 117.

Mr. Arnott alludes in a note to a case then under his observation of destructive disease of the right knee-joint consequent to delivery. Death not having taken place, the actual state of the veins is of course uncertain, and the case so far only goes to confirm the statements respecting the liability of parturient women to articular disease. The following passage is the only proof of the dependence of these affections on phlebitis which our author offers:—

"Upon the connexion of this affection of the joints in puerperal women with inflammation of the veins, there is an *appearance* of direct evidence in two of the cases related by M. Velpeau, in his paper on Phlegmasia Alba Dolens, although the description is somewhat incomplete. In one of these, death took place on the sixtieth day after labour, in the other, on the twenty-sixth, under great constitutional disturbance and exhaustion. In both, purulent matter was contained in the hypogastric and femoral veins. In the first case, the interpubic cartilage was softened, and pus was found in this situation; the same appearances were observed in the left sacro-iliac symphysis; the hip joint, also, contained purulent matter. In the second instance, the sacro-iliac and pubic symphyses were in a state similar to that just mentioned." 118.

It would be ridiculous to argue on a theory so fragile, on facts so feeble as those contained in the preceding paragraph. Mr. Arnott must surely intend to *hint* the connexion between phlebitis and those affections of the joints, and not to assert that he has proved it.

"The last circumstance to which I shall at present allude, is the occurrence in the puerperal state, of a disease of the eye, similar to that which took place in the instance of Dodging from the inflammation and suppuration in the vena saphena. It will be recollected that in this case the corneæ became opaque, the vessels of the conjunctivæ injected, and that after death, destructive changes were found within the globes. Now, although a remarkable, this is not an isolated instance of a peculiar disease of the eye succeeding to inflammation of a vein from mechanical injury." 118.

Our author accordingly details the case of Mr. Wardrop in which the jugular vein was found obliterated after the operation of tying the carotid and in which the left eye sloughed and collections of matter took place in several parts of the body. The case is too notorious to allow us to go over the ground again.

"When we consider the circumstances of this case, the venous hemorrhage, the constitutional disturbance, the formation of abscesses, and the appearances presented on dissection, and compare them with the consequences which have been observed to follow inflammation and suppuration of a vein, and the occurrences in the case of Dodging, can it be doubted that the affection of the eye in this instance, arose from the inflammation of the jugular vein, and from the entrance of an inflammatory secretion, probably pus, into the blood?

"A disease of the eye, similar to that observed in the two cases above mentioned, occurs in the puerperal state, and has been described by Dr. Marshall Hall, and Mr. Higginbottom, in a paper published in Vol. XIII. of the Society's Transactions, under the title of 'Cases of Destructive Inflammation of the Eye, and of Suppurative Inflammation of the Integuments occurring in the Puerperal state, and apparently from Constitutional causes.' In all of these cases, six in number, five of which came under their own observation, the affection of the eye took place in from five to eleven days after delivery. It was preceded and accompanied by serious indisposition, in every instance terminating fatally, and under symptoms of extreme exhaustion. The affection of the eye was characterized by redness of the conjunctiva, intolerance of light, and contracted pupil; rapidly followed by opacity of the cornea, and excessive chemosis. In four instances, dissolution took place before the coats of the eye gave way; in the two others, this occurred, during life: and in one, where the process was observed, by ulceration of the coats round the cornea. In both of these cases, the collapsed globe had healed over previous to death. In each instance only one eye was affected, and that the left; with the exception of the case communicated by Mr. Ward, where it does not appear which eye was the seat of disease. With the disease of the eye, there also took place an inflammation of the integuments, first observed on the hand, but on careful examination found in the inferior, as well as the superior extremities, and under which, matter quickly formed. In one case only, there was no such inflammation.

"The authors of this communication conjecture, that the morbid affection of the eye had a constitutional origin. No examination after death seems to have been made in any of these cases.

"Considering the circumstances under which the affection of the eye took place, its characters, and the deposition of pus under the integuments of the body; are we not justified, (on comparing these with the known consequences of inflammation of veins, and the frequency of this affection in those of the uterus after parturition,) in attributing such disease of the eye to inflammation of the uterine veins, and to the introduction of pus into the circulation?" 122.

We really must protest against this species of reasoning which if once admitted into medical writings would sap their genuine inductive character, and involve us in a greater tornado of theories and speculations than even blind our eyes and din our ears at present. Without one shadow of positive proof Mr. Arnott asks if we are not *justified* in attributing this affection of the eyes to inflammation of the uterine veins, and inquires if it *can be doubted* that it depended on the inflammation of the jugular in Mr. Wardrop's case. Yes, we answer boldly that the latter *may* be doubted and that we are *not* justified in pronouncing on the former. Had Mr. Arnott contented himself with pointing out the cases in question and remarking the certain degree of similarity between them, he would have stood on ground from which no one could dislodge him because he would have been under the cover of facts. But when on such slender premises he builds such wide assumptions, his position is so precarious that a feather would upset it. We should think that Mr. Arnott's craniological development must be large in the direction of *constructiveness*, for throughout the memoir and in the latter part of it especially he evinces remarkable talent for what is termed "framing" a theory. We admire the ingenuity but deplore the instability of the workmanship.

"Such are the facts which have induced me to conclude, that the inflammations and abscesses which arise in remote situations, after *injuries*, whether of the extremities or of the head, or after the *process of parturition*, are attributable to the existence of phlebitis in the part of the body primarily affected.

"In concluding these remarks, the object of which has been to point out the relation between the primary and secondary affections in phlebitis, and to establish the introduction of pus, or other inflammatory secretion, from the surface of the vein into the circulation, as the cause of the latter; I have not felt myself called upon to advance any opinion as to the manner in which this cause operates, in giving to some of the secondary affections their peculiar characters,—I allude more particularly to the depositions of pus and lymph, unattended by those changes in the texture of the parts, which usually precede the production of these fluids. I think it right, however, to state, that I must not be considered as regarding the matter so deposited to be actually that which has been brought into the circulation from the inflamed vein or veins. The disease of the eye, in which pus is not deposited, and the affection of the joints, exclusive of other considerations, clearly prove that the question is no longer one of a translation of matter merely, but one which involves the very difficult subject of the pathology of the blood, especially the share which diseased changes in this fluid have in the production of those phenomena which we are in the habit of comprehending under the term of inflammation." 123.

This concludes the memoir, a remarkable and talented one in many respects, and it now only remains to add a few words before parting with the author. It must have been seen already from the tenour of our remarks that we differ from him widely in several points, and think he has exceeded the strict and proper bounds of deduction in others. The paper may be divided into two parts, one embracing facts, the other theories, but both so intermingled, here merely dove-tailed and there amalgamated, that it is difficult to determine precisely the limits of each. We must say, however, that the facts collected by Mr. Arnott are extremely valuable and reflect the highest honour on his industry, research, and discrimination. They prove that phlebitis is a much more common disease than was imagined, that many of the notions of former authors respecting the mode in which it proved fatal, were erroneous, and that it gives a marked and undeniable tendency to produce consecutive and vicarious inflammations, if we may use the term, in other parts. But do the facts prove that phlebitis is the whole and sole cause, or even the most frequent one of visceral deposites after injuries, especially of the head, and operations? We believe that they do not, and the grounds for our belief or rather disbelief will be found in some measure scattered through the pages of this review.

Again with regard to our author's theories;—we dissent from him in toto respecting the exclusion of the agency of the nervous system, an exclusion that we consider a thousand times more absurd than he seems to think its admission would be. We also dissent from him on many minor points, but our space is too limited now to allow us to do more than again allude to the various incidental remarks and criticisms on which we have already ventured. We cannot say that we think much of the translation of pus, the *metempsychosis*, as it were, of our modern pathologists. The deposites, whether visceral, articular, or seated in the cellular substance, are neither in quality nor quantity related to the secretion of the parent wound or injured part, and it appears to us extremely ridiculous to imagine that suppurations beat up their quarters and take to another more favoured spot in the body, like a tribe of Bedouins in search of a spring in the desert, or an Arab palace under the magic influence of an Aladdin's lamp! To attribute these curious affections "to the very difficult subject of the pathology of the blood," is a supposition that leaves us and them where we were, and we believe after all that the theoretical notions respecting the visceral deposites are not one whit the clearer for the present investigation.

> "Well has he said, Athena's wisest son,
> "All that we know is—nothing can be known!"

We cannot take leave of the memoir without again expressing the high opinion we entertain of Mr. Arnott's industry and talents, qualities which we hope will be often applied to the elucidation of professional and scientific questions:—

> "Still from their cloistered walks to set them free,
> "And bring them to another Castalie."

